# Genomic Analyses of the Microsporidian *Nosema ceranae*, an Emergent Pathogen of Honey Bees

**DOI:** 10.1371/journal.ppat.1000466

**Published:** 2009-06-05

**Authors:** R. Scott Cornman, Yan Ping Chen, Michael C. Schatz, Craig Street, Yan Zhao, Brian Desany, Michael Egholm, Stephen Hutchison, Jeffery S. Pettis, W. Ian Lipkin, Jay D. Evans

**Affiliations:** 1 USDA-ARS Bee Research Lab, Beltsville, Maryland, United States of America; 2 Center for Bioinformatics and Computational Biology, University of Maryland, College Park, Maryland, United States of America; 3 Center for Infection and Immunity, Mailman School of Public Health, Columbia University, New York, New York, United States of America; 4 USDA-ARS Molecular Plant Pathology Laboratory, Beltsville, Maryland, United States of America; 5 454 Life Sciences/Roche Applied Sciences, Branford, Connecticut, United States of America; University of Melbourne, Australia

## Abstract

Recent steep declines in honey bee health have severely impacted the beekeeping industry, presenting new risks for agricultural commodities that depend on insect pollination. Honey bee declines could reflect increased pressures from parasites and pathogens. The incidence of the microsporidian pathogen *Nosema ceranae* has increased significantly in the past decade. Here we present a draft assembly (7.86 MB) of the *N. ceranae* genome derived from pyrosequence data, including initial gene models and genomic comparisons with other members of this highly derived fungal lineage. *N. ceranae* has a strongly AT-biased genome (74% A+T) and a diversity of repetitive elements, complicating the assembly. Of 2,614 predicted protein-coding sequences, we conservatively estimate that 1,366 have homologs in the microsporidian *Encephalitozoon cuniculi*, the most closely related published genome sequence. We identify genes conserved among microsporidia that lack clear homology outside this group, which are of special interest as potential virulence factors in this group of obligate parasites. A substantial fraction of the diminutive *N. ceranae* proteome consists of novel and transposable-element proteins. For a majority of well-supported gene models, a conserved sense-strand motif can be found within 15 bases upstream of the start codon; a previously uncharacterized version of this motif is also present in *E. cuniculi*. These comparisons provide insight into the architecture, regulation, and evolution of microsporidian genomes, and will drive investigations into honey bee–*Nosema* interactions.

## Author Summary

Honey bee colonies are in decline in many parts of the world, in part due to pressures from a diverse assemblage of parasites and pathogens. The range and prevalence of the microsporidian pathogen *Nosema ceranae* has increased significantly in the past decade. Here we describe the *N. ceranae* genome, presenting genome traits, gene models and regulatory motifs. *N. ceranae* has an extremely reduced and AT-biased genome, yet one with substantial numbers of repetitive elements. We identify novel genes that appear to be conserved among microsporidia but undetected outside this phylum, which are of special interest as potential virulence factors for these obligate pathogens. A previously unrecognized motif is found upstream of many start codons and likely plays a role in gene regulation across the microsporidia. These and other comparisons provide insight into the architecture, regulation, and evolution of microsporidian genomes, and provide the first genetic tools for understanding how this pathogen interacts with honey bee hosts.

## Introduction

Honey bees, *Apis mellifera*, face diverse parasite and pathogen challenges against which they direct both individual and societal defenses [1]. Severe honey bee colony losses have occurred in the past several years in the United States, Asia, and Europe. Some of these losses have been attributed to Colony Collapse Disorder (CCD), a sporadic event defined by high local colony mortality, the rapid depopulation of colonies, and the lack of known disease symptoms [2]. While causes of CCD are not yet known, and are likely to be multifactorial, increased pathogen loads in declining bees suggest a role for disease One candidate disease agent is the microsporidian *Nosema ceranae*, a species that has sharply increased its range in recent years [3]. Microsporidia are a highly derived lineage of fungi that parasitize a diverse assemblage of animals [4]. *N. ceranae* was first described from colonies of the Asian honey bee, *Apis cerana*, that were sympatric with *A. mellifera* colonies in China. Fries et al. [5] suggested that a host switch from *A. ceranae* to *A. mellifera* occurred relatively recently. Currently, *N. ceranae* is the predominant microsporidian parasite of bees in North America [6] and Europe [3].

*N. ceranae* is an obligate intracellular parasite of adult honey bees. Ingested spores invade the gut epithelium immediately after germination Intracellular meronts eventually lead to mesospores that can invade neighboring cells after host-cell lysis. Ultimately, hardier exospores are passed into the gut and excreted, at which point these exospores are infective to additional hosts. While congener *N. apis* appears to restrict its life cycle to the gut wall, *N. ceranae* was recently shown to invade other tissues [7]. Health impacts of *Nosema* infection on honey bees include a decreased ability to acquire nutrients from the environment and ultimately a shortened lifespan [8]. At the colony level, *Nosema* infection can lead to poor colony growth and poor winter survivorship. Nevertheless, *N. ceranae* is widespread in both healthy and declining honey bee colonies and its overall contribution to honey bee losses is debatable [9],[10],[11].

Genetic studies of *N. ceranae* and its infected host have been hindered by a lack of genetic data. Prior to this study, microsporidian sequence data were most extensive for the mammalian pathogen *Encephalitozoon cuniculi*, complemented by genome or EST surveys of several other human and insect pathogens [4],[12],[13],[14] and a recent draft annotation of the mammalian pathogen *Enterocytozoon bieneusi* [15]. Public sequences for *N. ceranae* were limited to ribosomal RNA loci. We therefore chose pyrosequencing to rapidly and cost-effectively characterize the *N. ceranae* genome, simultaneously illuminating the ecology and evolution of this parasite while enabling focused studies of virulence mechanisms and population dynamics. A genomic approach also leverages existing microsporidian and fungal genome sequence, advancing through comparative analysis our understanding of how microsporidian genome architecture and regulation has evolved. Microsporidia are remarkable in having small genomes that overlap prokaryotes in size, a propensity for overlapping genes and transcripts, few introns, and predicted gene complements less than half that found for yeast [16],[17],[18]. Microsporidian cells are also simplified at the organellar level and lack mitochondria, instead containing a genome-less organelle, the mitosome, that appears incapable of oxidative phosphorylation but may function in iron-sulfur biochemistry [19],[20]. Biochemical studies and sequence analyses have identified novel features of carbon metabolism and a dependency on host ATP, but much of their metabolism remains unclear [17],[19] and major metabolic pathways can differ substantially among species [15].

Here we analyze a draft genome assembly for *N. ceranae*, present a gene set of 2,614 putative proteins that can now be used to uncover salient aspects of *Nosema* pathology, and describe gene families and ontological groups that are distinct relative to other sequenced fungi. We provide formatted annotations for viewing with the Gbrowse genome viewer [21], which we hope will aid future studies of this economically important pathogen and of microsporidia in general.

## Materials and Methods

### *Nosema ceranae* spore purification

Honey bees infected with *N. ceranae* were collected from the USDA-ARS Bee Research Laboratory apiaries, Beltsville, MD. Alimentary tracts of these bees were removed and crushed in sterile water and filtered through a Corning (Lowell, MA) Netwell insert (24 mm diameter, 74 µm mesh size) to remove tissue debris. The filtered suspension was centrifuged at 3,000×g for 5 minutes and the supernatant discarded. The re-suspended pellet was further purified on a discontinuous Percoll (Sigma-Aldrich, St. Louis, MO) gradient consisting of 5 ml each of 25%, 50%, 75% and 100% Percoll solution. The spore suspension was overlaid onto the gradient and centrifuged at 8,000×g for 10 minutes at 4°C. The supernatant was discarded and the spore pellet was washed by centrifugation and suspension in distilled sterile water.

### Genomic DNA extraction

Approximately 10^6^ *N. ceranae* spores were suspended in 500 µl CTAB buffer (100 mM Tris-HCl, pH 8.0; 20 mM EDTA, pH 8.0; 1.4 M sodium chloride; 2% cetyltrimethylammonium bromide, w/v; 0.2% 2-mercaptoethanol) and broken by adding 500 µg of glass beads (425–600 µm, Sigma-Aldrich, St. Louis, MO) into the tube and disrupting the mixture at maximum speed for 2–3 minutes using a FastPrep Cell Disrupter (Qbiogene, Carlsbad, CA). The mixture was then incubated with proteinase K (200 µg/ml) for five hours at 55°C. Genomic DNA was extracted in an equal volume of phenol/chloroform/isoamyl alcohol (25∶24∶1) twice, followed by a single extraction in chloroform. The purified DNA was precipitated with isopropanol, washed in 70% ethanol, and dissolved in 50 µl sterile water. The concentration and purity of the DNA were determined by spectrophotometric absorption at 260 nm, and ratios of absorption at 260 nm and 280 nm.

### Sequencing and assembly

Extracted DNA was pooled, sheared, and processed using in-house protocols at 454 Life Sciences (Branford, CT). The template was then amplified by two separate runs of 32 emulsion-PCR reactions each, with each reaction comprised of templates containing 454-linker sequence attached to 600,000 sepharose beads [22]. Successful amplifications were sequenced using GS FLX picotiter plates and reads were trimmed of low-quality sequence before assembly with the Celera Assembler package CABOG [23].

### Gene predictions

Gene predictions were merged from three distinct sources. We first used the Glimmer package [24], which is designed for predicting exons of prokaryote and small eukaryote genomes, using a hidden Markov model to evaluate the protein-coding potential of ORFs. The model was initially trained on ORFs identified by Glimmer's *longorf* program, and then run with the following parameters: a minimum length of 90 codons, a maximum overlap of 50 bp, and a threshold score of 30. ORFs that contained a high proportion of tandem sequence repeats were ignored. Secondly, we identified all additional ORFs not predicted to be protein-coding by Glimmer that were BLASTX [25] matches to GenBank fungal proteins (at a lax expectation threshold of 1.0E-5). Finally, all remaining ORFs were searched with the HMMER program (http://hmmer.janelia.org) for Pfam-annotated protein domains [26] using an expectation threshold of 1.0×10E-1.

In 58 cases, adjacent ORFs matching different parts of the same GenBank protein or Pfam domain could be joined by hypothesizing a single-base frameshift error in the assembly. Our annotations span the start and stop codons of these conjoined ORFs and indicate the approximate site of the frameshift with the ambiguity characters N and X, respectively, in the nucleotide and protein sequence.

tRNA genes were predicted with the program ARAGORN [27]. Ribosomal genes were identified by BLASTN searches and alignments with existing *Nosema* ribosomal sequence in GenBank and the SILVA ribosomal database [28]. Nucleotide composition of protein-coding genes was investigated with the program INCA2.0 [29].

### Protein homology searches and functional annotation

We identified probable one-to-one orthologs among these three genomes using reciprocal best BLASTP matches, with the additional requirements that the best match have an expectation ≤1.0E-10 and 10^3^ lower than the second best match (identical protein predictions in *E. cuniculi* were considered equivalent). Best-fit homologs in yeast, as determined by BLASTP with a minimum expectation of 1.0E-10, were used to annotate *N. ceranae* genes with GO Slim ontologies [30]. Signal peptides were predicted using the SignalP 3.0 program [31] and transmembrane domains were predicted with TMHMM 2.0 [32]. Assignments to conserved positions in metabolic and regulatory pathways were based on the KEGG annotation resource [33], assisted by the Blast2Go program [34]. Repetitive elements were identified by searching against Repbase [35], by the pattern searching algorithm REPuter [36], and by intragenomic BLASTN analyses.

## Results

### Sequencing and assembly

Sequence information and annotations are posted in Genbank (www.ncbi.nlm.nih.gov) under Genome Project ID 32973. High-quality reads from two 454 GS FLX sequencing runs contributed 275.8 MB for assembly. The assembly was complicated by an extreme AT bias, frequent homopolymer runs (which are prone to sequencing error), and numerous repetitive elements (see below). Sixty-one independent assemblies were evaluated by systematically increasing the error parameter from zero to 6% in 0.1% increments. The final assembly used an error rate of 3.5% because this maximized both the N50 of contig size and the length of the longest contig. To search for potential mis-assembly, we compared this version to other assemblies using MUMmer [37]. We identified two contigs that likely contained collapsed repeats and replaced these with alternative versions assembled with a stricter error parameter. Other parameters of the assembly remained at their default CABOG settings. Sequencing and assembly statistics are summarized in **Table 1**.

**Table 1:** Statistics of draft *N. ceranae* genome assembly analyzed in this paper.

Stage	Category	Value*
Sequencing	High-quality reads	1,063,650
	High-quality bases	275,848,411
	Average high-quality read length	259.3
	Average high-quality read quality score	30.4
Assembly	Number of retained contigs	5,465
	Range of contig length (bp)	500–65,607
	Sum of contigs	7,860,219
	Contig N50 length	2,902
	Contig N50 number	470
	Average contig coverage	24.2

*: Sequence lengths in base pairs.

Accidental incorporation of non-target DNA sequence into genome assemblies is a ubiquitous hazard even with stringent sample preparation. We therefore used BLAST, depth of coverage, and G+C content as criteria to help identify potential contamination, but found no evidence of sequence derived from the host genome (*A. mellifera*), the sympatric congener *N. apis*, or another common fungal pathogen of bees, *Ascosphaera apis*. However, we did find evidence for low-level contamination by an unknown ascomycete fungus, indicated by generally short, low-coverage, high-GC contigs with consistently stronger BLASTX matches to Ascomycota than to Microsporidia. We therefore removed all contigs with less than five-fold coverage and a G+C content of 0.5 or greater (see **Fig. S1**), as well as any contig that matched ascomycete ribosomal or mitochondrial sequence.

After purging these suspect contigs and removing all contigs less than 500 bp in length, there remained 5465 contigs that totaled 7.86 MB of DNA. The N50 contig size of the pruned assembly was 2.9 kb (i.e., half of the total assembly, or 3.93 MB, was in contigs greater than 2.9 kb). The mean sequence coverage of contigs was 24.2×. Using the GigaBayes suite of programs [38],[39], we estimated the frequency of simple polymorphisms (indel or nucleotide, P≥0.90 per site) on the 100 longest contigs to be 1.0 per kilobase. Genomic G+C content of the final contig set was low compared with *E. cuniculi*, 26% vs. 47%, but typical of other surveyed microsporidia. Genomic contigs of *Enterocytozoon bieneusi* in GenBank have a G+C content of 24%, and Williams et al. [13] reported genomic G+C contents of *Brachiola algerae* and *Edhazardia aedis* to be 24% and 25%, respectively. Although several factors potentially associated with microbial base composition have been investigated, such as ambient temperature, mutation bias, and selection on genome replication rates, the causes of compositional bias remain unclear (see, for example, [40] and references cited therein).

Because genome assemblies may not accurately represent true genome size, due to such factors as redundancy at contig ends or collapsed repeats, we applied the method of Carlton et al. [41] to estimate genome size from sequence coverage, excluding repeats. We first classified all 22-mers occurring in the read sequence not more than 40 times as the unique portion of the genome. Using this filter, the average coverage was 26.6× and 28.2× for regions of at least 1 kb and 10 kb in length, respectively. The total length of the *N. ceranae* reads is 261.0 MB after filtering reads with G+C content higher than 50%. With these values, the total genome size could be as high as 9.8 MB. However, this G+C filter may be overly permissive; increasing the filter stringency to 35% G+C reduces the genome size estimate to 8.6 MB. An additional consideration is that, at the estimated level of coverage, we expect the entire genome to be sequenced with few singletons or small contigs. Yet 30.0 MB of read sequence assembled into contigs with 10 or fewer reads, including 5.5 MB of single-read contigs. These small contigs are likely to be from reads with relatively high sequencing error. If so, this would boost the average coverage of the assembly by 3×–3.5× and reduce the genome size to as low as 7.7 MB. Our attempts to measure the genome size empirically with pulse-field gel electrophoresis did not adequately resolve *N. ceranae* chromosomes. However, this technique in other *Nosema* species has yielded genome size estimates of 7.4–15 MB [42]. Thus, while our computational estimate is in reasonable agreement with current genome size estimates for the genus, an unknown but potentially significant portion of the genome may be unrepresented in this assembly and the absence of particular sequences should not be considered definitive.

### Sequence repetition

The genome sequence of *E. cuniculi* revealed an unusual distribution of sequence repeats, characterized by a lack of known transposable elements, a paucity of simple repeats, and an abundance of near-perfect segmental duplications of 0.5–10 kb in length. Pulse-field gel electrophoretic studies have identified gross variation in the size of homologous chromosomes among and within isolates of *E. cuniculi* [43] and the microsporidian *Paranosema grylli* [44], indicating that large segmental duplications are potentially important sources of intraspecific variation. The origins and gene content of such duplications are therefore of particular interest. While the present assembly limits our ability to describe larger segmental duplications in *N. ceranae*, we were able to investigate sequence repetition in the genome by searching for microsatellite motifs and by using REPuter [36] to detect complex repeats. All eight dinucleotide repeats found were ‘AT’ repeats, ranging from a perfect 9-unit repeat to an imperfect (3 mismatches) 21-unit repeat. There were six AAT repeats greater than 6 units in length and four ATC repeats. We confined our search for complex repeats to those contigs greater than 1,200 bp in length, so as to identify repeats likely to be dispersed in the genome rather than confined to the most poorly assembled fragments. REPuter identified a total of 4,731 sequence pairs with at most three mismatches that ranged from 70 bp (the minimum threshold for detection) up to 312 bp in length with a median of 85 bp. Repeats were over-represented on smaller contigs, even within the analyzed set of relatively long contigs, indicating that they had affected assembly success.

BLASTN analyses of the REPuter-identified repeats against the *N. ceranae* genome revealed a novel dispersed repeat with a conserved core domain approximately 700 bp in length (**Fig. S2**). The boundaries of the element are not completely clear because the conserved domain often occurs as tandem copies, there are two or more subtypes of the element based on multiple sequence alignments, and, as expected, copies are most abundant on short contigs and near contig ends. Using an E-value cutoff of 1.0E-5, we identified one or more matches on 250 contigs. No conserved coding potential was evident for these elements, nor did we detect any homology with sequences in GenBank or Repbase. Surprisingly, this element contains a candidate polII promoter that is well conserved and generally scores between 0.90 and 1.00 (the maximum value) when submitted to a neural network prediction tool [45]. Whether this promoter-like motif is functional and, if so, whether it produces a coding or noncoding transcript remain to be seen. However, it is clear from BLAST searches that this promoter sequence is not associated with any of our predicted genes (see below), nor could we identify it in *E. cuniculi* or yeast.

### Predicted genes and associated features

We identified 2,614 putative protein-coding genes, with reference names, coordinates, and annotation features provided in **Text S1**. Gene models were not required to have a start methionine to allow for gene predictions truncated at ends of contigs and (rarely) the possibility of non-canonical start codons or frameshifts in the assembly. In addition to BLAST-hit annotations, **Text S1** also lists Pfam protein domains as well as signal peptide and transmembrane motifs. **Texts S2** and **S3**, respectively, contain GFF-formatted data and a configuration file for viewing our annotations with the Gbrowse viewer [21]. An example of these annotations viewed in GBrowse is shown in **Fig. S3**.

The number of protein-coding genes we have predicted for *N. ceranae* lies in between the 1,996 Refseq proteins given by GenBank for the sequenced *E. cuniculi* genome and the 3,804 predicted for *E. bieneusi* from sequence representing only two-thirds of the estimated genome content. The density of genes on the 100 largest *N. ceranae* contigs averaged 0.60 genes/kb (64.8% coding sequence). This is a lower proportion of coding sequence than found in *E. cuniculi* and *Antonospora locustae* (0.94 and 0.97 genes/kb, respectively [4]), but comparable to some other microsporidia [13]. However, gene density declines considerably with contig size (**Fig. S4**), consistent with a preponderance of repetitive elements (described above and to follow) or other noncoding sequence in these regions.

We found forty-six contigs containing sequences that matched *N. ceranae* ribosomal sequence at an expectation of E<1.0E-10, but no contig contained a complete ribosomal locus. Assembly of Sanger-sequenced *Enterocytozoon bieneusi* genomic DNA resulted in a similar fracturing of ribosomal sequence [15]. It seems likely, then, that *N. ceranae* ribosomal loci contain abundant polymorphism and/or error-prone sequences that are recalcitrant to our assembly parameters. Polymorphism among rRNA loci has been reported for *N. bombi* [46] whereas the sub-telomeric location of *E. cuniculi* ribosomal loci [16] suggests potential a association with repetitive sequence.

We found 65 tRNA genes with 44 distinct anticodons (**Fig. S5**), sufficient to match all codons with third-position wobble. Five tRNA gene predictions contain introns, and a putative tRNA intron-endonuclease (NcORF-01478) was also identified. Aminoacyl-tRNA synthetases were found for all twenty standard amino acids, as well as two enzymes involved in selenoamino acid metabolism (NcORF-00234 and NcORF-00337). tRNA genes are particularly abundant for the common amino-acid leucine (11 genes), yet there was no overall correlation between the frequency of an amino-acid in predicted proteins and the number of tRNAs for that amino-acid (not shown). In comparison, there are only 46 tRNA genes in *E. cuniculi*, yet they also match 44 distinct anticodons. *E. cuniculi* contains a tRNA matching the codon ‘CCC’ that was not found in *N. ceranae*, whereas *N. ceranae* contains a tRNA with the anti-codon TCA that is not found in *E. cuniculi*. tRNAs of this latter type match the stop codon TGA and are assumed to be charged with the nonstandard amino-acid selenocysteine [47]. This tRNA was also predicted by tRNAscan-SE [48], and alignment of the raw reads revealed no evidence of sequence ambiguity in the anticodon loop. Elongation factors for selenocysteine incorporation are uncharacterized outside of mammals and bacteria (reviewed in [49]). We did not find a homolog of the SelU family [50], the most broadly distributed selenoprotein family across eukaryotes. Of the six selenoproteins reported by the SelenoDB database [49] to be present in yeast, we found only two clear homologs in *N. ceranae*, NcORF-01193 and NcORF-01194, both of which are glutathione peroxidases. These genes do not appear to be selenoproteins in *N. ceranae* because the predicted stop codons are not TGA and no downstream coding potential is evident.

**Figure 1:**
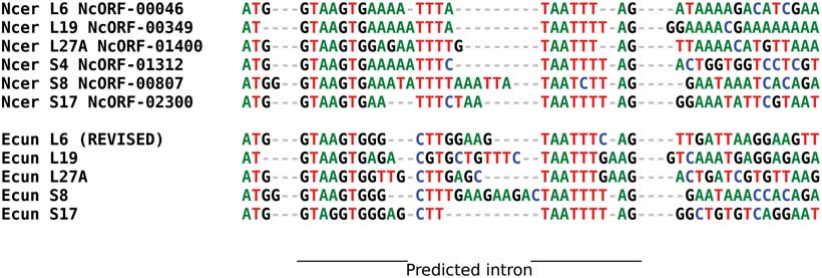
Alignment of the 5′ region of six ribosomal protein genes in *N. ceranae* that contain predicted introns, together with the five orthologs of these genes in *E. cuniculi*. Alignment begins with the start codon, which is interrupted by an intron in the ribosomal protein L19. The gene model for the *E. cuniculi* L6 protein was modified based on comparison with the *N. ceranae* nucleotide sequence and translation. Introns were manually aligned to illustrate regions of sequence conservation. Ncer = *N. ceranae*, Ecun = *E. cuniculi*.

We identified six genes with predicted short introns as well as two spliceosomal proteins, the U1- and U6-associated proteins (NcORF-00067 and NcORF-01581). While Katinka et al. [16] inferred eleven ribosomal protein genes with introns in *E. cuniculi*, only five of the *N. ceranae* orthologs also contained an intron. The sixth *N. ceranae* gene containing an intron encodes the S4 ribosomal protein, which lacks an intron in *E. cuniculi*. The intronic sequences show fairly strong conservation within and between species (**Fig. 1**), suggesting selection for efficient recognition by the spliceosomal machinery.

Only one non-ribosomal, protein-coding gene of *E. cuniculi* has been predicted to contain introns (gi|107906965, a phosphatidyltransferase; [16]). Alignment with the *N. ceranae* and yeast orthologs (not shown) indicates that while the *N. ceranae* gene (NcORF-02116) may have a single intron in a position similar to the second of two predicted *E. cuniculi* introns, it can be read through giving an equally plausible translation. Given the lack of other known introns in non-ribosomal proteins, it seems more parsimonious to conclude that the *E. cuniculi* sequence contains an assembly error or nonsense mutation. This interpretation is consistent with analysis of the more-distantly related *E. bieneusi* [15], in which no introns were found in ribosomal proteins and potential introns identified in non-ribosomal proteins could be read through without detriment to their alignment with *E. cuniculi* homologs. Thus, spliceosomal introns in microsporidia appear to be both dispensable and, when present, confined to a particular ontological group, although confirmation of this hypothesis will require analysis of full-length cDNAs.

### Regulatory motifs

Patterns of transcript initiation and termination vary dramatically among microsporidians, and common eukaryotic regulatory motifs appear to have been obscured by genome compaction [14],[51]. To characterize the 5′ context of *N. ceranae* coding sequences, we analyzed a sample of 280 genes with one-to-one orthologs in *E. cuniculi* and yeast that align well at the 5′ end (to maximize our confidence in the predicted start methionine). Plotting the frequency of the yeast TATA box motif, TATA[AT]A[AT] [52], in the 200-bp region upstream of the start codon shows a pronounced peak in motifs that begin near the −27 position, relative to their frequency in random sequence of the same base composition (**Fig. S6**). The proportion of upstream sequences (12.5%) with a TATA motif beginning within the window −25 to −32 is three-fold greater than the randomly generated sample (4.1%). These data suggest that TATA-like promoters, which occur in about 20% of yeast genes [52], are also important components of *N. ceranae* gene regulation. In comparison, an unspecified AT-rich transcription initiation sequence was identified within 120 bases of *E. cuniculi* ORFs [16]. Phylogenetic footprinting and/or direct experiment may enable a more sensitive model of microsporidian promoters.

**Figure 2:**
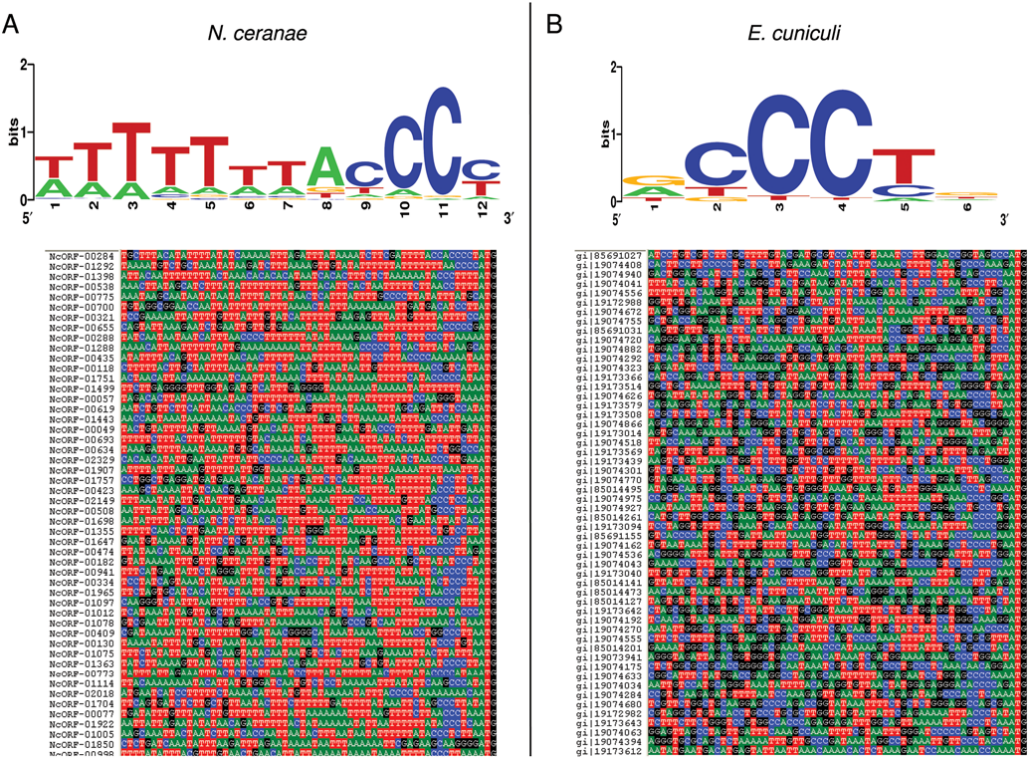
Conserved sense-strand motif upstream of start codons of *N. ceranae* and *E. cuniculi* genes. A. Sequence logo of motif identified by MEME and representative unaligned sequence upstream of the start codon (the last three positions) of *N. ceranae* genes. B. Sequence logo of motif identified by MEME and representative unaligned sequence upstream of the start codon of *E. cuniculi* genes. See text for details.

**Figure 3:**
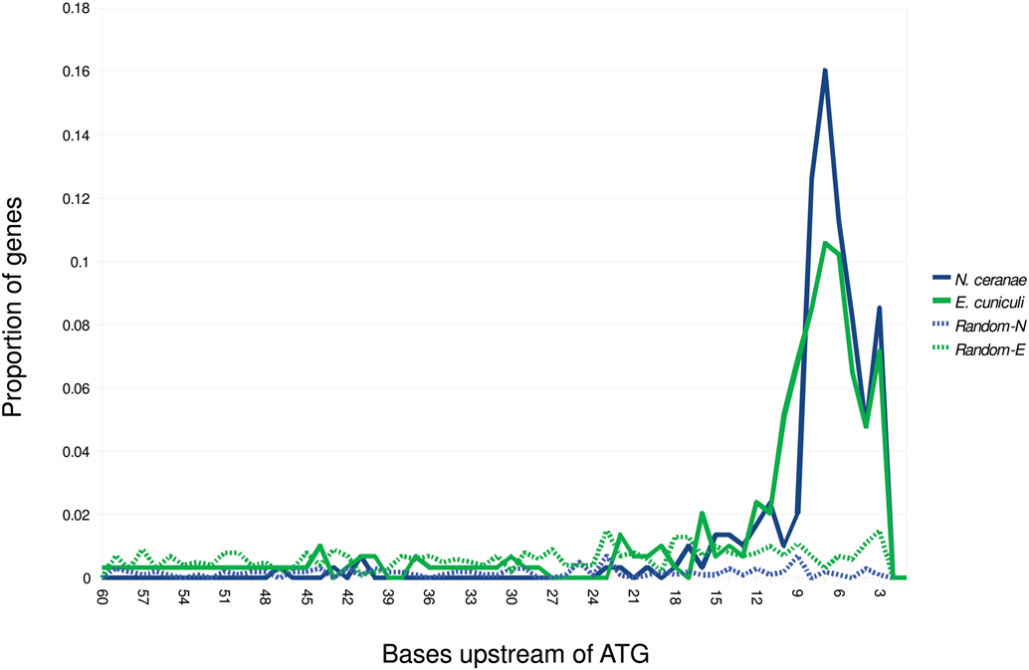
Over-representation of the nucleotide sequence ‘CCC’, common to both motifs shown in Figure 2, in the region upstream of start codons. The horizontal axis is the distance from the start codon in bases (5′ to 3′ with the ATG not shown at the 3′ end), and the vertical axis is the proportion of genes in the sample (n = 292, see text) in which the first base of a cytosine triplet occurs that number of bases upstream of the start. Both genomes show a much higher frequency of CCC occurring close to the start codon than do random sequences of the same length and base composition (“N-random” and “E-random” refer to the base composition of *N. ceranae* and *E. cuniculi* sequences, respectively). For x-axis values of 1 or 2, the frequency is zero by definition.

In addition to analyzing TATA motifs, we searched for novel 5′ motifs by applying MEME [53] to a slightly expanded version of the reference set described above (n = 292), by narrowing the search region to 60 positions upstream of the start codon. MEME identified a motif containing a cytosine triplet that is highly over-represented (E = 1.9E-258), and these motifs occur predominantly within 15 bp of the start codon. The motif is further characterized by a thymine homopolymer just upstream of the cytosine triplet. A sequence logo and an alignment of representative sequences are shown in panel A of **Fig. 2**. We then used MEME to identify the single highest-scoring motif in the equivalent regions of *E. cuniculi* (panel B of **Fig. 2**). A shorter but otherwise similar motif was identified, consisting of a cytosine triplet bracketed by a purine and a pyrimidine, as is the case in *N. ceranae*. However, the expectation for a motif of this length and composition in the *E. cuniculi* sample was 9.2E+7, i.e. it is statistically invisible in the absence of the corroborating *N. ceranae* motif. Because the cytosine triplet is the most conserved component of the two motifs, we investigated the distribution of cytosine triplets nearest the start codon. We compared these distributions in both genomes relative to random sequence of the same base composition. The distributions of cytosine triplets were highly concordant between the two genomes, and much more frequent than expected by chance between the −10 and −3 positions, as shown in **Fig. 3**.

### Codon use and amino-acid content

**Figure 4:**
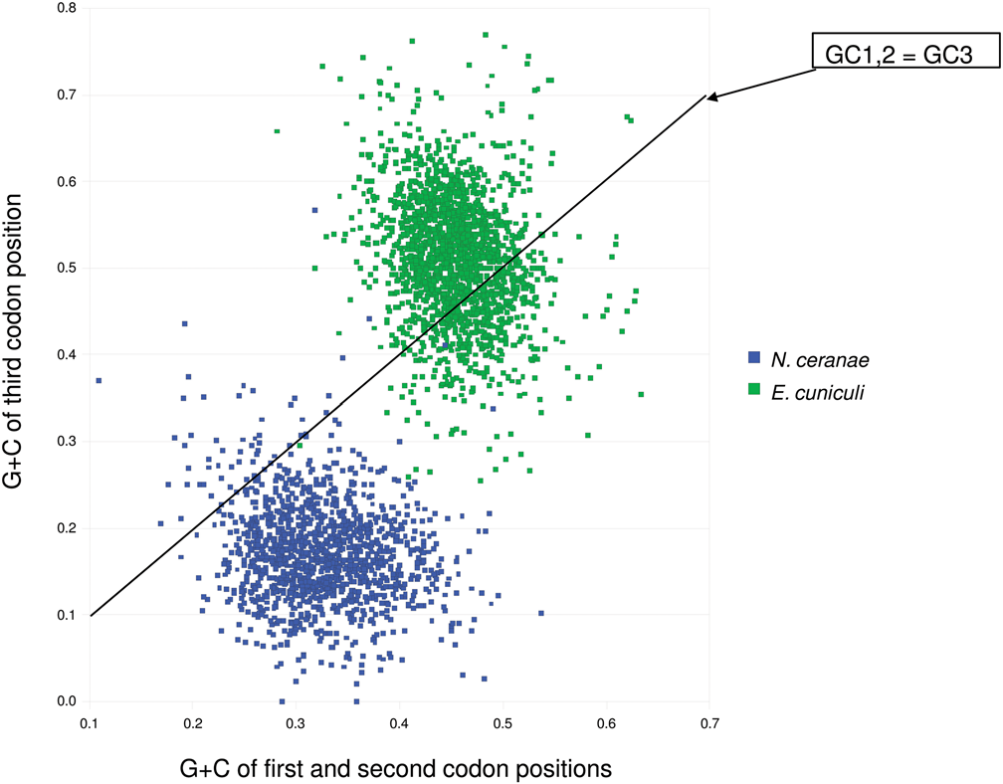
G+C content of *N. ceranae* and *E. cuniculi* genes. The horizontal axis is the proportion of G+C in the first two codon positions (GC1,2), whereas the vertical axis represents the G+C content of the third position (GC3). Substitutions at the third position are usually synonymous and thus are expected to reflect mutational equilibrium in the absence of other evolutionary forces such as codon bias. The two genomes overlap very little in G+C content of genes, and differ more at the third position than at the first two positions.

Considering the high AT-content of the *N. ceranae* genome, the preponderance of AT-rich synonymous codon use, particularly relative to *E. cuniculi*, is unsurprising (**Fig. S7**). More remarkable is that the G+C content of *N. ceranae* genes (27%) is only minimally higher than the genome average of 26%, primarily because third position G+C is substantially lower (18%) than the intergenic average of 23% (**Fig. 4**). *E. bieneusi* genes downloaded from GenBank (January 2009) show an even greater bias toward A+T in the third position (25% genic and 12% third position G+C), suggesting that this may be a common feature of AT-rich microsporidia. However, few *N. ceranae* genes showed significant codon-usage bias after controlling for length and nucleotide composition (**Fig. S8**), and the most codon-biased genes in *N. ceranae* appear unrelated by homology or function to the most biased genes in the other microsporidian samples.

Divergence in nucleotide composition between *N. ceranae* and *E. cuniculi* has also impacted nonsynonymous sites, as it has in other organisms [54],[55]. Pooling the proteomes of the two species reveals differences in amino-acid use (**Fig. S9**, panel A) that are consistent with conservative substitutions driven by mutation pressure. For example, *E. cuniculi* encodes more arginine and less lysine than does *N. ceranae*, suggesting conservative replacement of lysine codons AAA and AAG with arginine codons AGA and AGG. Other notable differences in *N. ceranae* relative to *E. cuniculi* include higher frequencies of isoleucine, phenylalanine, and tyrosine (amino-acids coded by AT-rich codons) and lower frequencies of glycine and alanine, which have GC-rich codons. The magnitude of these differences are only slightly attenuated when considering only the core set of orthologs both species share with yeast (panel B of **Fig. S9**) rather their complete proteomes, demonstrating that even ancient proteins participating in conserved cellular processes are impacted by evolutionary change in base composition.

### Ortholog comparisons with other microsporidia

Estimates of orthology based on reciprocal BLAST scores (**Table 2**; see Materials and Methods) indicate that 1,252 *N. ceranae* genes (47.9%) are one-to-one orthologs with *E. cuniculi* proteins. *N. ceranae* shares about twice as many genes with *E. cuniculi* as either shares with *Saccharomyces cerevisiae*, and we identified only 411 one-to-one orthologs that are conserved among all three genomes, or seven percent of the already streamlined yeast proteome (5880 predicted proteins). To identify conserved microsporidian-specific genes that may be relevant to their life history, we searched all *N. ceranae* – *E. cuniculi* ortholog pairs by BLASTP against the GenBank nr database and against Pfam. We identified 11 genes (**Fig. S10**) that had no Pfam domain significant at E<0.5 and no BLAST hit outside the phylum Microsporidia with an expectation below 1.0E-5.

**Table 2:** Estimates of the number of *N. ceranae* genes that are homologous or orthologous to genes in the microsporidian *E. cuniculi* and yeast.

Species	Gene category*	Relative to *E. cuniculi*	Relative to *S. cerevisiae*
*N. ceranae*	Homologous	1,366	700
	Orthologous	1,252	466
*E. cuniculi*	Homologous		740
	Orthologous		511

*: See text for assignment method.

Only one of these conserved proteins (NcORF-00083) is homologous to a known polar tube protein, PTP3 [56]. The polar tube proteins are major structural components of the microsporidian polar filament/polar tube, a defining character of the phylum that enables invasion of the host cell [57]. Two additional polar tube components have been identified in other species, PTP1 and PTP2 [58]. The genes encoding these proteins are closely linked in *E. cuniculi* and *A. locustae* but are divergent at the amino-acid level [59]. As all three PTPs have signal peptides, we searched our *N. ceranae* gene predictions for adjacent genes with this motif. NcORF-01664 and NcORF-01663 encode two such proteins and have similar lengths and amino-acid compositions to PTP1 and PTP2, respectively, of *E. cuniculi* and *A. locustae* (**Fig. 11**), and are likely the orthologous genes. Yet they share only 16.7% and 19.6% percent identity, respectively, when aligned with their putative *E. cuniculi* orthologs. These results further confirm the low sequence conservation among putatively orthologous polar tube proteins [59], suggesting that they may be evolving rapidly in response to host variation.

We examined the pattern of synteny between *N. ceranae* genes and *E. cuniculi* genes on the three longest contigs (∼170 kb). Syntenic regions encompassing 2–10 genes are common on these contigs (**Fig. S12**), although changes in order and orientation within these regions are also common. This conservation of synteny appears to be somewhat higher than that reported between *E. cuniculi* and *E. bieneusi* [60], consistent with a much higher level of protein-sequence conservation in general between *N. ceranae* and *E. cuniculi* homologs than between either species and *E. bieneusi*. To illustrate this, we performed a BLAST search with the set of *N. ceranae* genes that have one-to-one orthologs in yeast and *E. cuniculi* against the combined GenBank protein set for *E. bieneusi*, *E. cuniculi*, and *S. cerevisiae*. **Figure S13** shows the distribution of BLAST scores and expectations for the best match between *N. ceranae* and each of the other three genomes (only results for *N. ceranae* proteins with matches in all three genomes are plotted, *n* = 234). The *E. cuniculi* match was substantially better in almost all cases, whereas the *E. bieneusi* match was, on average, only modestly better than the *S. cerevisiae* match. The smaller proportion of the *E. bieneus*i genome (relative to *E. cuniculi*) that has been sequenced [15] represents a potential bias in this comparison, because there is a greater likelihood that a higher-scoring *E. bieneusi* homolog exists that has not been annotated, yet such a bias could not explain the broad and consistent pattern shown in **Fig. S13**. A ribosomal phylogeny of microsporidia places *N. ceranae* and *E. cuniculi* closer to each other than to *E. bieneusi* with 100% bootstrap support, yet all three species are in the same subclade of the five microsporidian subclades identified by Vossbrinck and Debrunner-Vossbrinck [61]. Thus, our data would seem to support the conclusion of Keeling and Slamovits [62] that the rate of protein-sequence evolution in microsporidia is high relative to the pace of change in genome organization.

We identified seven pairs of adjacent *N. ceranae* genes that overlap by 4–11 bp. All of these gene predictions were supported by BLAST and/or Pfam matches, and aligned with *E. cuniculi* homologs, when identified, at the overlapping ends. Furthermore, six of the seven gene pairs were oriented in opposite directions and overlapped at their 3′ ends (**Fig. S12**), eliminating the possibility of artifacts arising from a misidentified start codon. The seventh pair was arranged in a tail-to-head configuration, but we could not identify a plausible alternative start codon that would eliminate the overlap. Short overlaps of adjacent ORFs were reported for a comparable number of genes in *E. cuniculi* and *E. bieneusi* [15], yet the identity of the genes involved is not conserved, suggesting that overlaps arise independently in each lineage and do not strongly constrain gene-pair synteny. For example, only four of the seven overlapping pairs of *N. ceranae* genes are adjacent in *E. cuniculi* (and they are non-overlapping).

A large number of genes in surveyed microsporidians are either weakly conserved among microsporidians or novel. The proportion of *N. ceranae* proteins lacking a BLASTP match to *E. cuniculi* at a threshold expectation of 1.0E-5 was 42.8% (1,119/2,614). The proportion of *E. bieneusi* genes lacking homologs in *E. cuniculi* was reported to be 82.8% (3,151/3,804) [15]. While a nontrivial fraction of gene models are expected to be false predictions and legitimate genes may escape annotation, these sources of error seem insufficient to account for the apparent scale of gene turnover among microsporidians. Furthermore, in both *N. ceranae* and *E. cuniculi*, the complement of potentially novel genes includes large multi-gene families within a single species, a phenomenon that cannot be explained by chance occurrence of long, non-coding ORFs. In *E. cuniculi*, for example, there are over fifty members of a well-conserved protein family, formalized by the Pfam domain DUF2463 and constituting 4% of the *E. cuniculi* proteome, that has not been reported in any other taxon. The ‘interB’ group of genes [63] is another large multi-gene family (∼30 members) identified in *E. cuniculi* for which homologs have been detected in only two other microsporidian genera. In *N. ceranae*, we identified five multi-gene families with at least ten members (**Text S4**) for which we also failed to detect homology in the GenBank, Pfam, or Repbase databases. Several considerations suggest that these *N. ceranae* gene families may in fact be uncharacterized transposable elements. Almost all of these genes were predicted on short contigs of 500–1000 bp, with only one contig (Nc00042) containing members of a novel family (NcORF-00713 and NcORF-00714) together with known microsporidian genes (NcORF-00705 - NcORF-00711). In a few cases, genes are very similar at the nucleotide level, including synonymous sites, and this similarity extends to adjacent downstream noncoding sequence. Furthermore, two additional multi-gene ‘families’ (**Text S4**) that are also found only on small contigs appear to be distinct members of the *Merlin* transposon superfamily [64]. Unfortunately, given the small contigs on which they occur, we cannot systematically compare the complete coding sequence of these genes in their genomic context, so as to characterize associated noncoding sequences and/or insertion-sites. Note that these contigs are unlikely to be contaminants because most have coverage comparable to the assembly average.

Regardless of whether these novel proteins are encoded by ‘host’ genes or are transposable elements, they represent a significant fraction of coding sequence and suggest that forces of genome expansion are active in some microsporidian lineages, as noted for other species [13],[14]. Moreover, we also observed many unambiguous examples of transposable elements of various classes, samples of which are shown in **Text S5**. Highly significant matches to Pfam domains and Repbase annotated sequences include *gypsy*-type LTR retrotransposons previously identified in another *Nosema* species, *N. bombycis* [65]. The most common coding elements, however, appear to be DNA transposons such as *Merlin*, *Helitron*, *piggyBac*, and *MULE*. Although these classes of repetitive element are widespread in eukaryotes, including other pathogens (e.g., [66]), their diversity and activity in microsporidia promise to be fertile areas of research, given the current paradigm of selection-driven genome compaction, the greater facility by which intron-less host genes may be transposed by active elements, and the opportunity for lateral transfer of elements between intracellular parasites and their hosts. Nonetheless, the longest polyA stretch we identified within 150 bases downstream of a gene prediction was only 12 bases (two occurrences), suggesting that retrogenes derived from reverse-transcribed mRNA are infrequent in *N. ceranae*.

### Functional annotation

**Figure 5:**
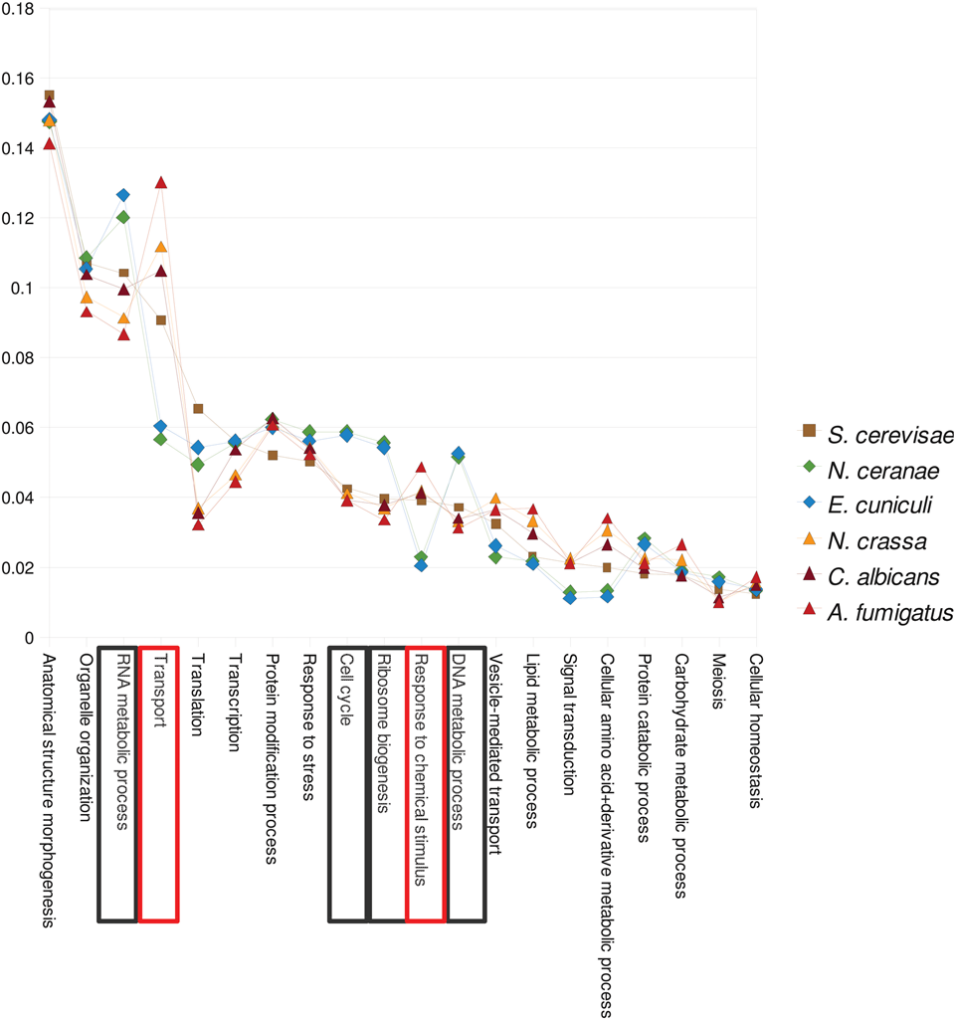
The twenty most abundant ontology terms of the GO Slim classification for yeast, *S. cerevisiae*, in descending order and compared with the abundance of these terms applied to five other genomes as described in the Materials and Methods. Lines are used instead of points in order to highlight the variation among species. The microsporidians *N. ceranae* and *E. cuniculi* differ concordantly from the four fungal genomes, which together represent a diversity of phylogenetic positions and life-history strategies, including facultative pathogens. The concordance of the microsporidian annotations imply that common ancestry and/or shared life-history traits have shaped the allocation of their genomes to various functional categories. Terms in black boxes are notably more common in the microsporidia, whereas terms in red boxes are notably less common. Note that for all species the frequencies sum to one and therefore are not independent.

As an initial functional characterization of the *N. ceranae* proteome, we assigned to each *N. ceranae* protein the GO Slim terms [30] of its closest yeast homolog. For comparison, we used the same method to re-annotate the proteomes of *E. cuniculi* and three other sequenced fungi with a range of life histories: *Neurospora crassa*, *Aspergillus fumigatus*, and *Candida albicans*. **Figure 5** shows the twenty most common yeast GO Slim terms for biological processes, ranked by their frequency among all such annotations, and the frequency of these terms in annotations assigned to the other species. *E. cuniculi* and *N. ceranae* differ from the fungal genomes in a concordant fashion, indicating a strong signal of phylogeny and life history at this coarse-grained level of functional annotation. Gill et al. [14] also identified a strong similarity of ontological groups between a sample of *E. aedis* ESTs and *E. cuniculi* genes.

We identified candidate constituents of a mitosome (**Table 3**) by examining proteins with GO terms that mapped to mitochondrial-specific processes other than those involved in the maintenance and expression of a mitochondrial genome (for details see [16],[19],[20]). We also searched for homologs of mitosome components identified in other microsporidia [16],[20]. We identified three proteins involved in protein import and refolding, a mitochondrial Hsp70 and homologs of the yeast proteins Erv1 and Cpr3. We did not find clear homologs of other membrane transport proteins (TOM70, TIM22, TOM40, and Imp2) that were reported for *E. cuniculi*, however, suggesting a simplified membrane structure or transport process in *N. ceranae* mitosomes. We found homologs of five proteins involved in iron-sulfur/redox biochemistry (ferredoxin, Nfs1, Grx5, frataxin, and superoxide dismutase) as well as seven mitochondrial ABC transporters, which export products of iron-sulfur biogenesis to the cytoplasm. The presence of numerous homologs of yeast mitochondrial proteins that function in iron/sulfur metabolism further supports the centrality of this process in the mitosome. Like *E. cuniculi* [16] and *A. locustae* [20], *N. ceranae* has homologs of G3PDH, NADH-cytochrome b5 reductase, and both pyruvate dehydrogenase subunits, although where these enzymes function in microsporidian metabolism remains to be clarified. Finally, we also found a homolog of Puf3, which has roles in mitochondrial control and maintenance.

**Table 3:** *N. ceranae* predicted proteins that are candidate constituents of a mitosome.

Category	*N. ceranae* protein	Yeast homolog name	GenBank description
Protein import/export	NcORF-00033	Atm1	Mitochondrial inner membrane ATP-binding cassette (ABC) transporter, exports mitochondrially synthesized precursors of iron-sulfur (Fe/S) clusters to the cytosol
	NcORF-00145	Atm1	
	NcORF-00146	Atm1	
	NcORF-00663	Atm1	
	NcORF-00705	Atm1	
	NcORF-00710	Atm1	
	NcORF-00711	Atm1	
	NcORF-00832	Cpr3	Mitochondrial peptidyl-prolyl cis-trans isomerase (cyclophilin), catalyzes the cis-trans isomerization of peptide bonds N-terminal to proline residues; involved in protein refolding after import into mitochondria
	NcORF-01760	Ecm10	Heat shock protein of the Hsp70 family, localized in mitochondrial nucleoids, plays a role in protein translocation, interacts with Mge1p in an ATP-dependent manner; overexpression induces extensive mitochondrial DNA aggregations
	NcORF-00417	Nfs1	Cysteine desulfurase involved in iron-sulfur cluster (Fe/S) biogenesis; required for the post-transcriptional thio-modification of mitochondrial and cytoplasmic tRNAs; essential protein located predominantly in mitochondria
	NcORF-00673	Ynr070	Putative transporter of the ATP-binding cassette (ABC) family, implicated in pleiotropic drug resistance; the authentic, non-tagged protein is detected in highly purified mitochondria in high-throughput studies
Iron/sulfur biochemistry	NcORF-00350	Erv1	Flavin-linked sulfhydryl oxidase of the mitochondrial intermembrane space (IMS), oxidizes Mia40p as part of a disulfide relay system that promotes IMS retention of imported proteins; ortholog of human hepatopoietin (ALR)
	NcORF-00677	Sod2	Mitochondrial superoxide dismutase, protects cells against oxygen toxicity; phosphorylated
	NcORF-01511	Frataxin	Frataxin, regulates mitochondrial iron accumulation; interacts with Isu1p which promotes Fe-S cluster assembly; interacts with electron transport chain components and may influence respiration; human homolog involved in Friedrich's ataxia
	NcORF-01632	Ferredoxin	Ferredoxin of the mitochondrial matrix required for formation of cellular iron-sulfur proteins; involved in heme A biosynthesis; homologous to human adrenodoxin
	NcORF-01638	Grx5	Hydroperoxide and superoxide-radical responsive glutathione-dependent oxidoreductase; mitochondrial matrix protein involved in the synthesis/assembly of iron-sulfur centers; monothiol glutaredoxin subfamily member along with Grx3p and Grx4p
Other	NcORF-01880	Puf3	Protein of the mitochondrial outer surface, links the Arp2/3 complex with the mitochore during anterograde mitochondrial movement; also binds to and promotes degradation of mRNAs for select nuclear-encoded mitochondrial proteins

## Discussion

*Nosema ceranae* is fast becoming a worldwide pathogen of western honey bees [3],[6], and this pathogen has been implicated in losses of individual bees, colonies, and local populations [8],[67]. *N. ceranae* apparently moved from its original host, *Apis cerana*, to sympatric *A. mellifera* populations in Asia [5], and has since dispersed globally over the last decade with help from trade in honey bees or their products. The present work provides essential tools for better understanding the mechanisms and outcomes of infection by *N. ceranae*.

An immediate benefit of genome sequencing is the insight gained from comparative analyses with genetic model organisms and other related species. For example, differences in the proportion of the genome devoted to specific ontological groups can suggest factors that underlie adaptations to intracellular parasitism. Applying the standardized vocabulary of the Gene Ontology Consortium [30], it is clear that *N. ceranae* and *E. cuniculi* differ from yeast and other fungi in a highly concordant fashion, devoting a smaller fraction of their genome to transport and response to chemical stimuli, and a larger fraction to growth-related categories. These trends are consistent with adaptations during microsporidian evolution to a life cycle buffered from environmental variation but requiring rapid reproduction. Although this comparison suggests a degree of constancy in the complement of genes microsporidians have retained from their common ancestor with yeast, it is clear that surveyed species differ by more than just a handful of host-specific genes. Instead, a large portion of their proteomes is poorly conserved at the sequence and/or gene-content levels, at least among genera. Important variation in metabolic capabilities has also become apparent [15]. As more species are surveyed, the degree and nature of genes that are shared will become much clearer. For example, factors such as transmission mechanisms, the presence or absence of vacuolar membranes around meronts, and karyon type [42], as well as host characteristics such as body temperature, may well correlate with genomic composition.

Since nucleotide substitutions that generate phenotypic variation can occur in regulatory as well as coding sequence, the identification of sequence features potentially associated with gene regulation is of particular interest. Comparative genomics can provide such insights and is especially valuable for organisms that cannot be cultured or manipulated *in vitro*. For example, the start-associated motif we identified may be an important regulator of transcription in *N. ceranae*, and can be a focus of comparative studies of microsporidian transcript structure, or analyzed experimentally in transformed yeast cells. Further comparative studies of microsporidian gene regulation could help explain why some species have apparently lax control of transcription resulting in a high frequency of run-on transcripts [51].

A further advantage of genome sequencing accrues from the thousands of potential genetic polymorphisms in the sequenced isolate that may be useful for population genomics. In particular, patterns of diversity and recombination can uncover important clues regarding the life history and dispersal of these enigmatic parasites. Outstanding questions include the relationships among *N. ceranae* strains infecting *A. cerana* versus *A. mellifera*, whether there is an association between *N. ceranae* genetic markers and the severity of disease, and the degree of sexual reproduction.

In summary, honey bees face myriad pathogens and parasites, and there is longstanding documentation of the negative effects of *Nosema* species on bee health. The characterization of the *N. ceranae* genome presented here should advance our understanding and eventual mitigation of *Nosema* parasitism. The identification of conserved and novel genes and domains is an initial step in unraveling the many enigmatic aspects of *Nosema*-honey bee interactions. Of particular interest are the 89 gene models encoding signal peptides, as these proteins are candidate secretory proteins that may interact with host tissue. Ongoing efforts to describe the genome of congener *N. apis* may uncover sequence features correlated with the proposed host switch and rapid spread of *N. ceranae* into *A. mellifera* colonies. In a broader context, this work contributes to an emerging capacity to study the genomic scale of interactions between honey bees and the host of diverse, co-occurring pathogens that they face.

## Supporting Information

Text S1 Predicted coding genes and annotations.
(5.08 MB XLS)

Text S2 GFF-formatted file of annotations.
(2.90 MB ZIP)

Text S3 Configuration file for Gbrowse.
(0.003 MB ZIP)

Text S4 Fasta file of aligned members of novel families of predicted proteins, and two families of Merlin transposons.
(0.02 MB ZIP)

Text S5 Selected aligments of *N. ceranae* contigs with annotated transposable elements in Repbase.
(0.01 MB ZIP)

Figure S1 G+C content (horizontal axis) versus coverage (vertical axis) of *N. ceranae* contigs. Panels are >1000 bp, 500–1000 bp, and <500 bp, left to right. Note wide range of mean coverage, even for large contigs.
(2.13 MB TIF)

Figure S2 Partial sequence alignment of copies of a novel dispersed repeat found on 250 contigs using conservative BLAST criteria. The conserved sequence includes a candidate polII promoter but no long ORF.
(7.68 MB TIF)

Figure S3 Screenshots of annotated *N. ceranae* assembly viewed with the Gbrowse application.
(4.23 MB TIF)

Figure S4 Table illustrating the progressive decline in gene density as contig size decreases.
(0.88 MB TIF)

Figure S5 The 65 *N. ceranae* tRNA genes predicted by ARAGORN [27], ordered by the corresponding amino-acid.
(1.65 MB TIF)

Figure S6 Sense-strand matches to the yeast TATA motif, TATA[AT]A[AT], in the 200-bp region upstream of high-confidence start codons. The vertical axis shows the proportion of all matches upstream of the sampled genes (n = 280, see text) that begin at the specified distance from the start codon. There is a pronounced spike in TATA box motifs occurring in the vicinity of the −27 position relative to their frequency in random sequence of the same base composition.
(1.42 MB TIF)

Figure S7 Codon usage of *N. ceranae* (red) and *E. cuniculi* (green) genes, plotted using INCA [29]. Each bar represents the proportion of all codons encoding a given amino-acid that are the specified codon. Thus, the values are one by definition for the single-codon amino-acids, tryptophan and methionine.
(5.78 MB TIF)

Figure S8 Codon bias of genes of three microsporidian genomes. Only *N. ceranae* genes with homology to genes in *E. cuniculi* are plotted. Vertical axis is ENC' (Novembre JA [2002] Accounting for background nucleotide composition when measuring codon usage bias. Mol Biol Evol 19: 1390–1394), a measure of codon bias adjusted for nucleotide composition, plotted versus third-position G+C (GC3). Few *N. ceranae* genes have an ENC' less than 50. Those that do are not obviously related, by homology or ontology, to comparably biased genes in the other two species. Thus, strong codon bias may not be a useful predictor of gene-expression level in microsporidia as it is in a variety of other microbes.
(1.22 MB TIF)

Figure S9 Frequency of each amino-acid, indicated by single-letter codes, in predicted proteins of *N. ceranae* and *E. cuniculi*. A. The frequency of each amino-acid in those genes that have one-to-one orthologs in the other microsporidian genomes and yeast. The conservation of these genes suggests that they have essential and ancient functions. B. The frequency of each amino-acid in all predicted proteins of the indicated species.
(1.93 MB TIF)

Figure S10 Characteristics of ‘microsporidian-specific’ genes, orthologous pairs of genes found in *N. ceranae* and *E. cuniculi* that lack apparent homology with proteins of taxa outside of order Microsporidia.
(1.12 MB TIF)

Figure S11 Amino-acid compositions of the putative polar tube proteins PTP1 and PTP2 in *N. ceranae* and two other microsporidians, *E. cuniculi* and *A. locustae*. Lengths of predicted proteins, in amino acid residues, is given in parentheses.
(1.86 MB TIF)

Figure S12 Degree of synteny between the *N. ceranae* contigs and *E. cuniculi* chromosomes. For each of the three largest contigs, predicted *N. ceranae* genes are shown in order along the contig (not to scale). The relative orientation of each gene is indicated by the arrow. *N. ceranae* genes shaded gray have one-to-one orthologs with *E. cuniculi* genes, whereas circled genes have homologs in *E. cuniculi* but not a one-to-one ortholog. Unmarked genes have no detected homolog in *E. cuniculi* (see text). The position in kilobases and relative orientation of the *E. cuniculi* ortholog is shown directly below the *N. ceranae* gene in the row corresponding to its chromosomal location. Coordinates are based on the GenBank record for each chromosome. These contigs contain regions of extensive, coarse-scale synteny with *E. cuniculi*, within which there can be considerable change in gene order or orientation. There are also numerous breaks in synteny associated with either a switch in *E. cuniculi* chromosome or an intervening, non-homologous gene.
(2.06 MB TIF)

Figure S13 Relative sequence conservation between *N. ceranae* proteins and their homologs in other reference species. *N. ceranae* genes with one-to-one orthologs in *E. cuniculi* and yeast (see text) were BLASTP searched against the combined proteomes of *E. cuniculi*, *E. bieneusi*, and yeast. The number of *N. ceranae* genes with high-scoring matches in all three reference species in this data set was 234. The upper panel plots the BLASTP score of each *N. ceranae* gene versus the best match in each species, ordered along the X-axis by descending score versus *E. cuniculi*. Values are represented as lines rather than points for easier visualization. The lower panel plots the BLASTP expectation (E-value) in ascending order versus *E. cuniculi*. E-values equal to zero were set to 1.0E-200 to allow a logarithmic scale.
(2.63 MB TIF)

Figure S14 Alignment of adjacent *N. ceranae* genes that are supported by homology (see Results) and that overlap in sequence. Each set of three sequences represents a contig and two adjacent genes. Start and stop codons are indicated by boxes.
(2.37 MB TIF)
