# Supplementary figures and images for: Genomic Analyses of the Microsporidian Nosema ceranae, an Emergent Pathogen of Honey Bees
Source: PLoS Pathog. 2009 Jun 5;5(6):e1000466. doi: 10.1371/journal.ppat.1000466 (PMC2685015; doi:10.1371/journal.ppat.1000466)

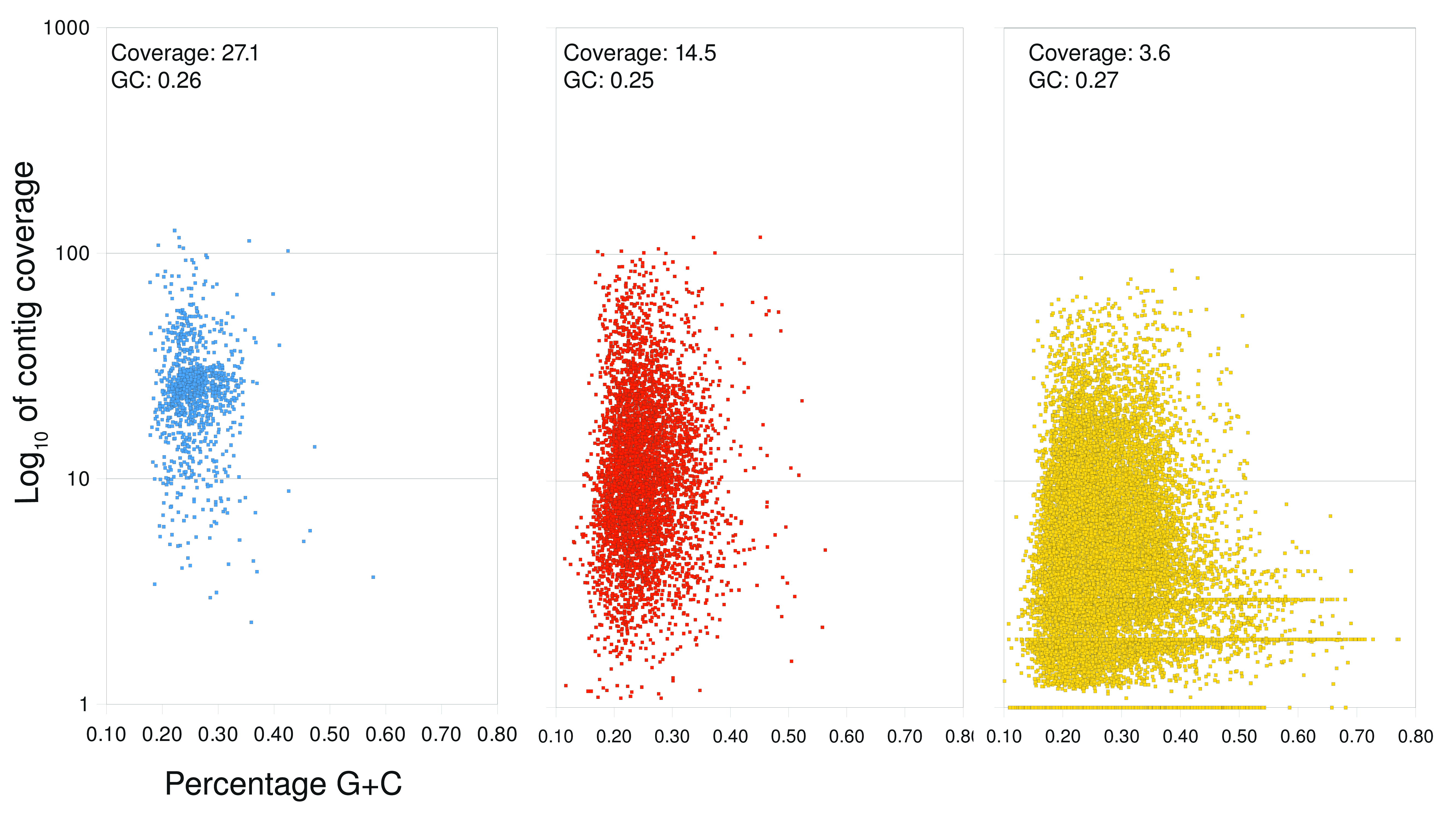

Supplement: Figure S1 — G+C content (horizontal axis) versus coverage (vertical axis) of N. ceranae contigs. Panels are >1000 bp, 500–1000 bp, and <500 bp, left to right. Note wide range of mean coverage, even for large contigs. (2.13 MB TIF) [file ppat.1000466.s006.tif]

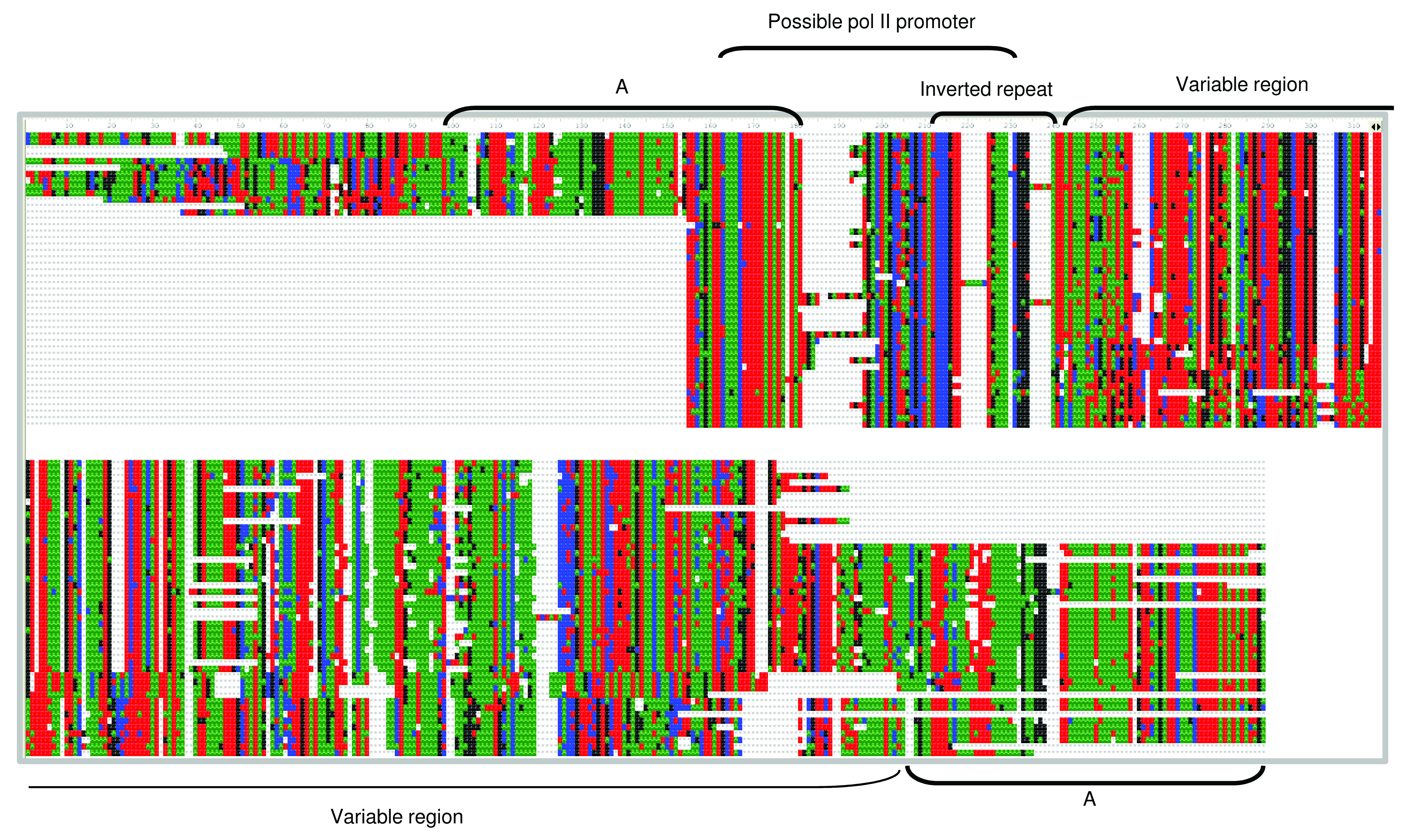

Supplement: Figure S2 — Partial sequence alignment of copies of a novel dispersed repeat found on 250 contigs using conservative BLAST criteria. The conserved sequence includes a candidate polII promoter but no long ORF. (7.68 MB TIF) [file ppat.1000466.s007.tif]

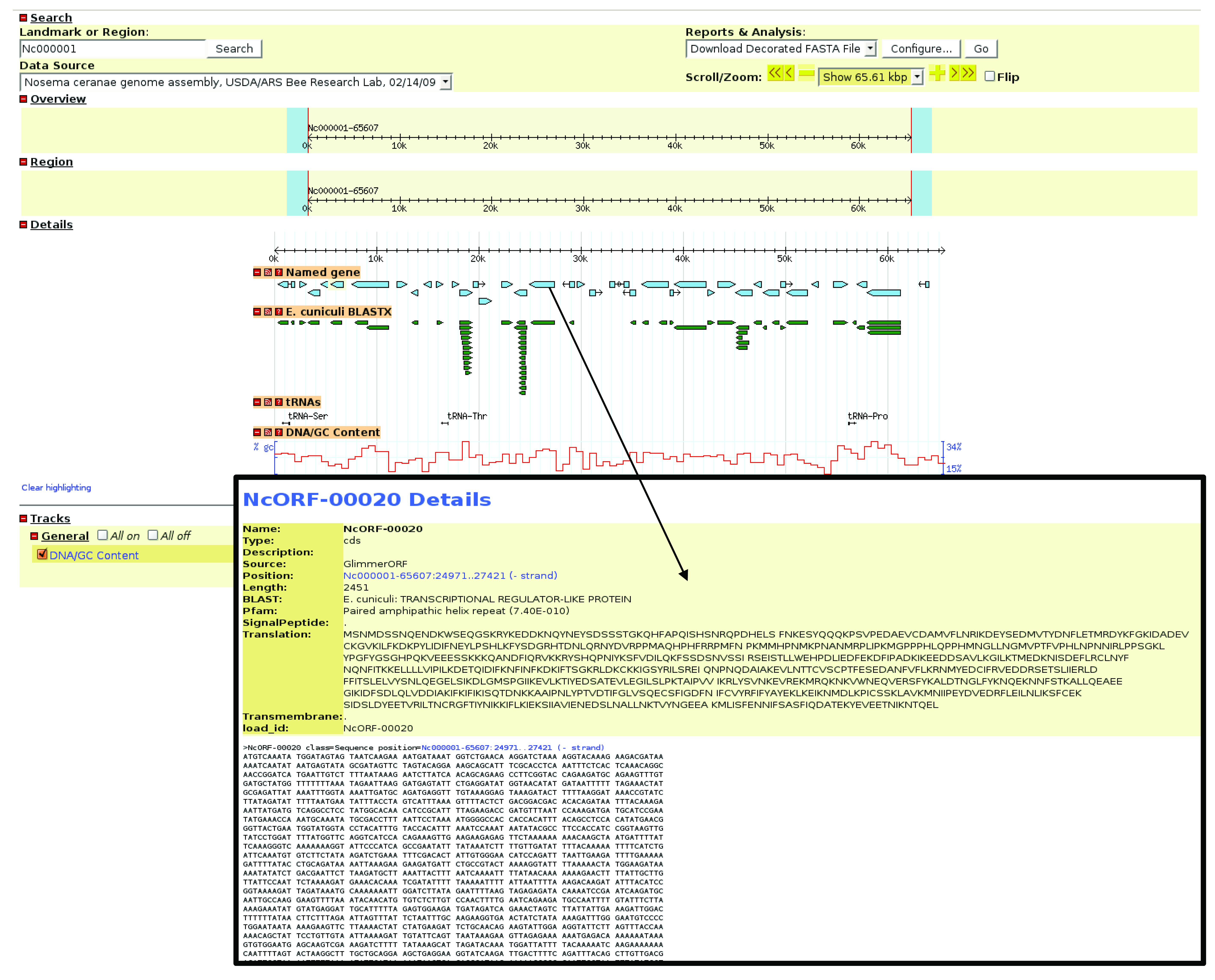

Supplement: Figure S3 — Screenshots of annotated N. ceranae assembly viewed with the Gbrowse application. (4.23 MB TIF) [file ppat.1000466.s008.tif]

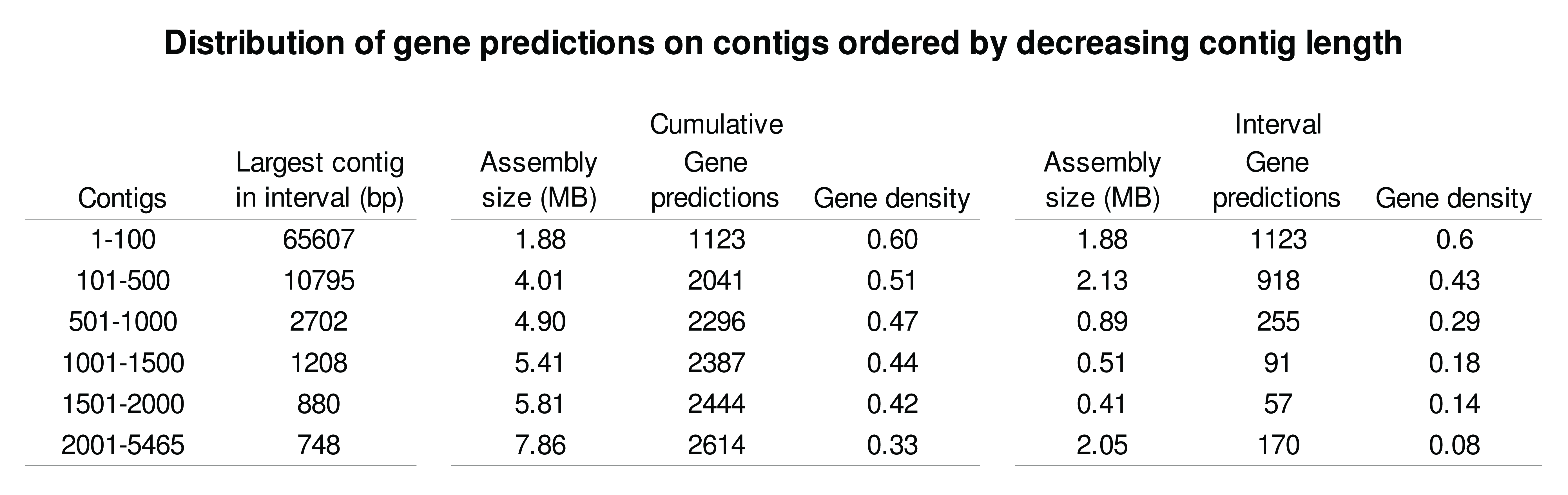

Supplement: Figure S4 — Table illustrating the progressive decline in gene density as contig size decreases. (0.88 MB TIF) [file ppat.1000466.s009.tif]

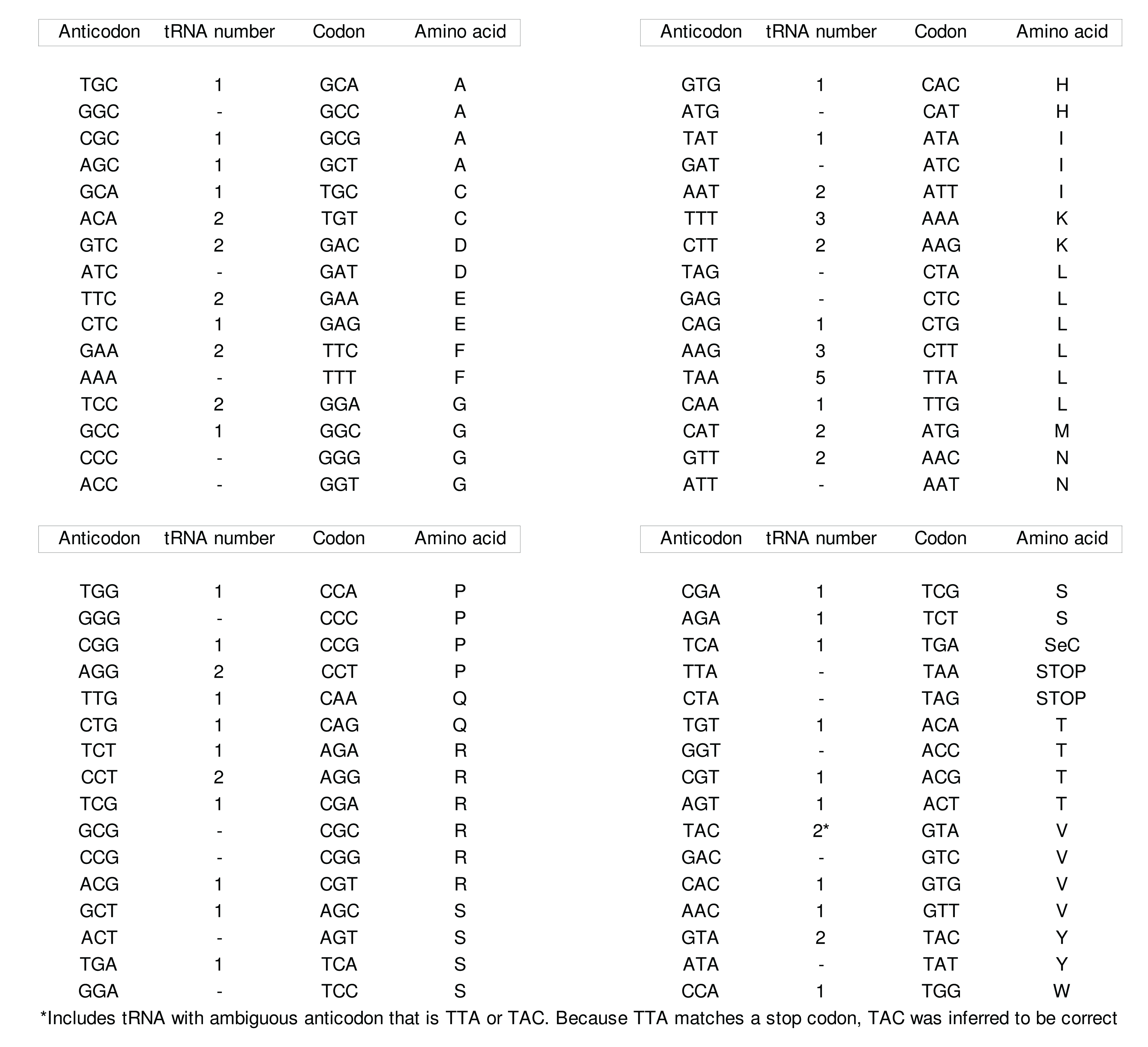

Supplement: Figure S5 — The 65 N. ceranae tRNA genes predicted by ARAGORN [27], ordered by the corresponding amino-acid. (1.65 MB TIF) [file ppat.1000466.s010.tif]

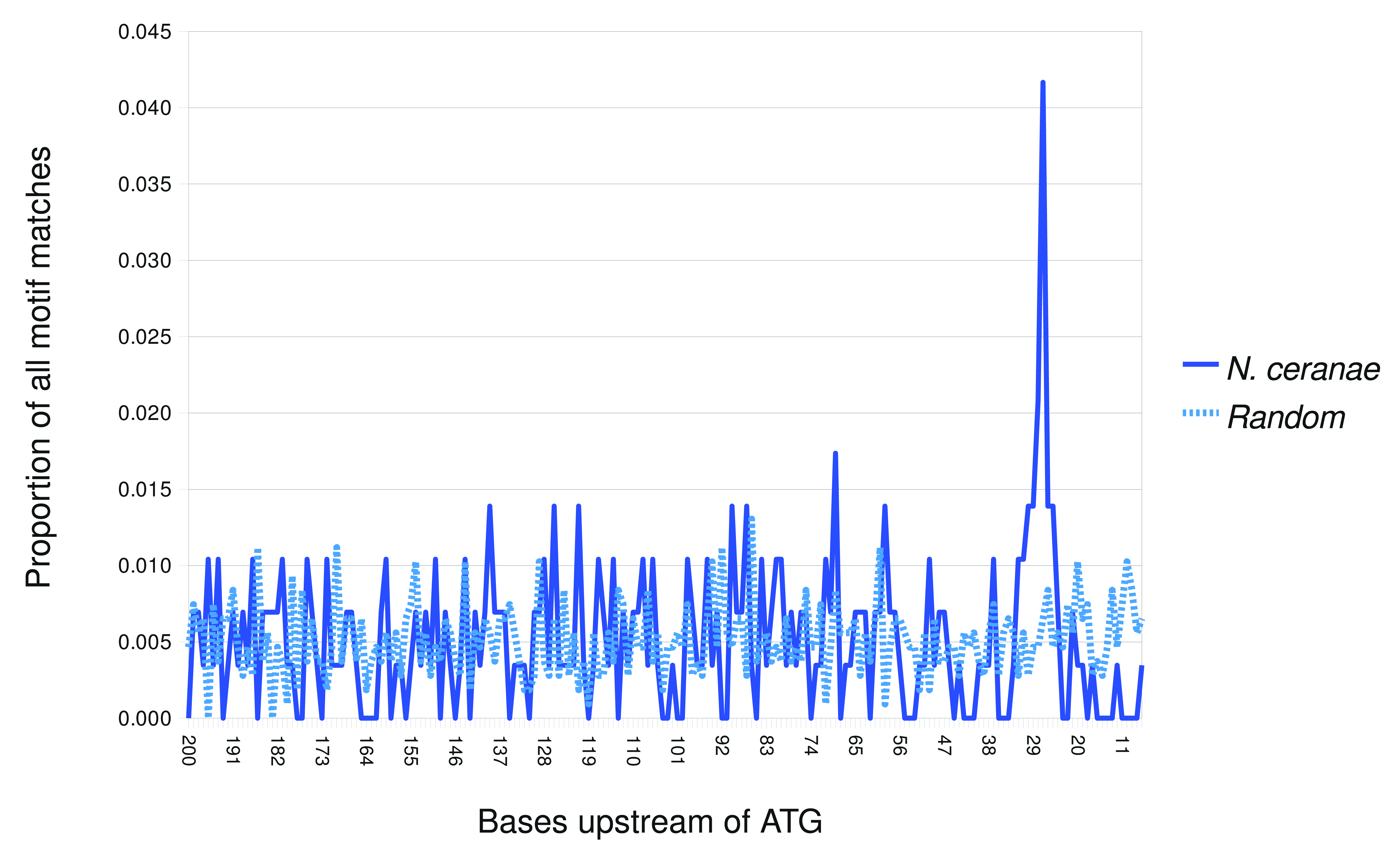

Supplement: Figure S6 — Sense-strand matches to the yeast TATA motif, TATA[AT]A[AT], in the 200-bp region upstream of high-confidence start codons. The vertical axis shows the proportion of all matches upstream of the sampled genes (n = 280, see text) that begin at the specified distance from the start codon. There is a pronounced spike in TATA box motifs occurring in the vicinity of the −27 position relative to their frequency in random sequence of the same base composition. (1.42 MB TIF) [file ppat.1000466.s011.tif]

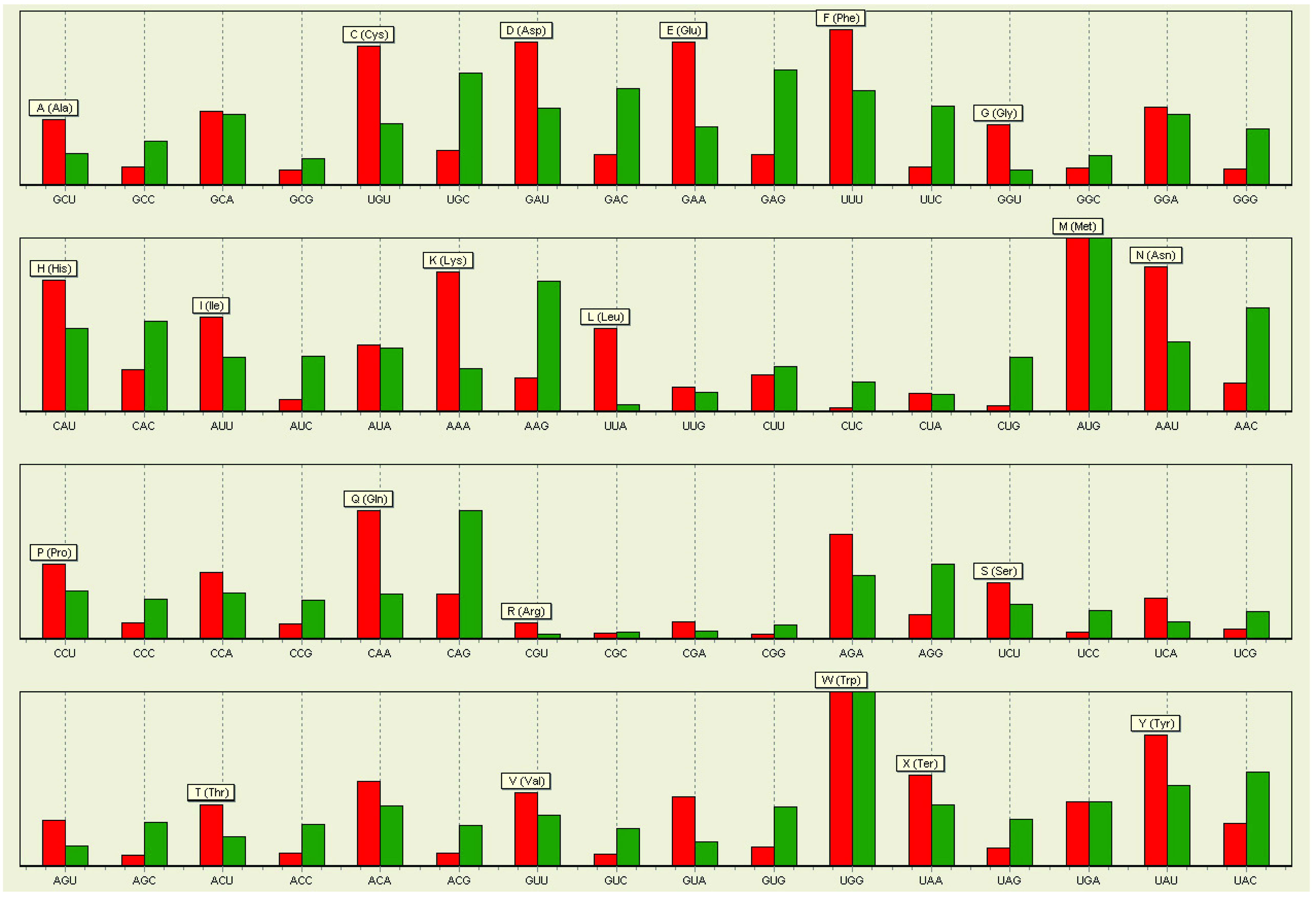

Supplement: Figure S7 — Codon usage of N. ceranae (red) and E. cuniculi (green) genes, plotted using INCA [29]. Each bar represents the proportion of all codons encoding a given amino-acid that are the specified codon. Thus, the values are one by definition for the single-codon amino-acids, tryptophan and methionine. (5.78 MB TIF) [file ppat.1000466.s012.tif]

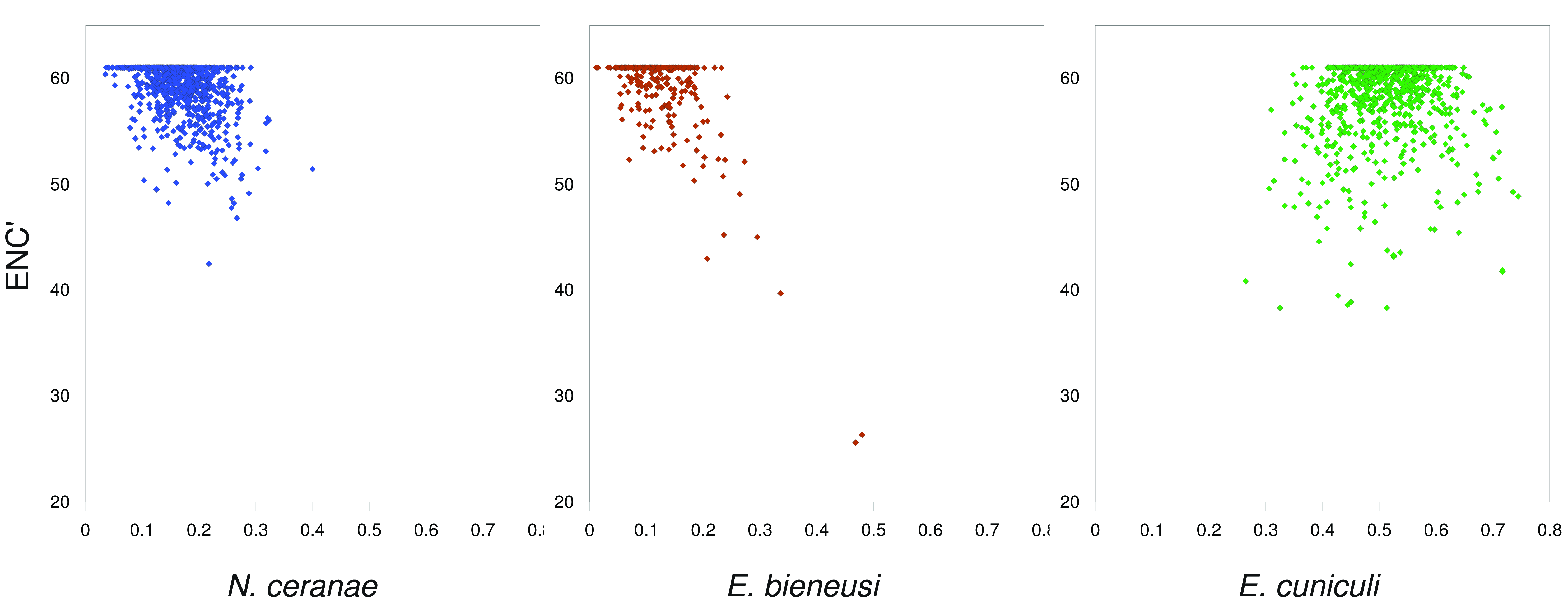

Supplement: Figure S8 — Codon bias of genes of three microsporidian genomes. Only N. ceranae genes with homology to genes in E. cuniculi are plotted. Vertical axis is ENC' (Novembre JA [2002] Accounting for background nucleotide composition when measuring codon usage bias. Mol Biol Evol 19: 1390–1394), a measure of codon bias adjusted for nucleotide composition, plotted versus third-position G+C (GC3). Few N. ceranae genes have an ENC' less than 50. Those that do are not obviously related, by homology or ontology, to comparably biased genes in the other two species. Thus, strong codon bias may not be a useful predictor of gene-expression level in microsporidia as it is in a variety of other microbes. (1.22 MB TIF) [file ppat.1000466.s013.tif]

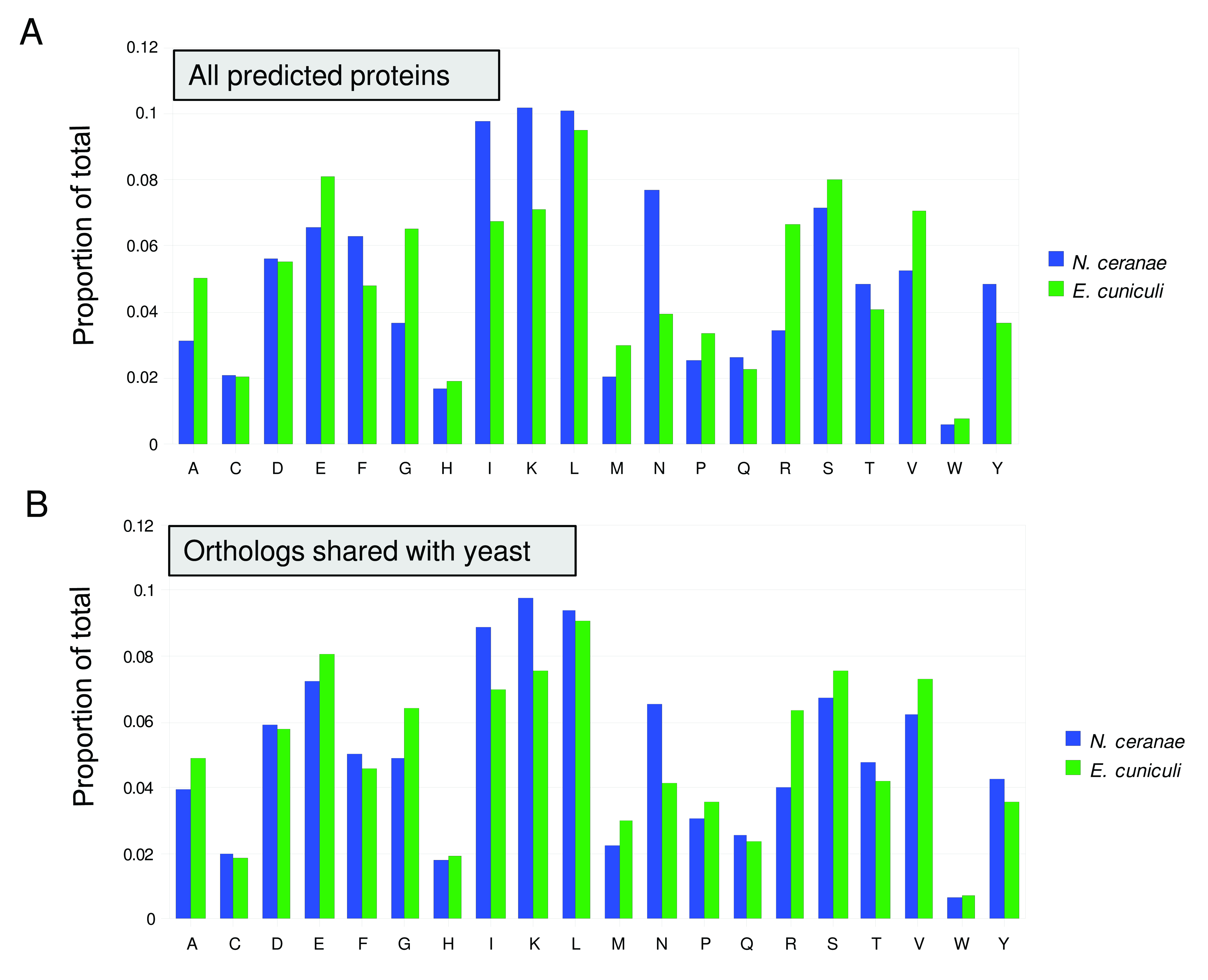

Supplement: Figure S9 — Frequency of each amino-acid, indicated by single-letter codes, in predicted proteins of N. ceranae and E. cuniculi. A. The frequency of each amino-acid in those genes that have one-to-one orthologs in the other microsporidian genomes and yeast. The conservation of these genes suggests that they have essential and ancient functions. B. The frequency of each amino-acid in all predicted proteins of the indicated species. (1.93 MB TIF) [file ppat.1000466.s014.tif]

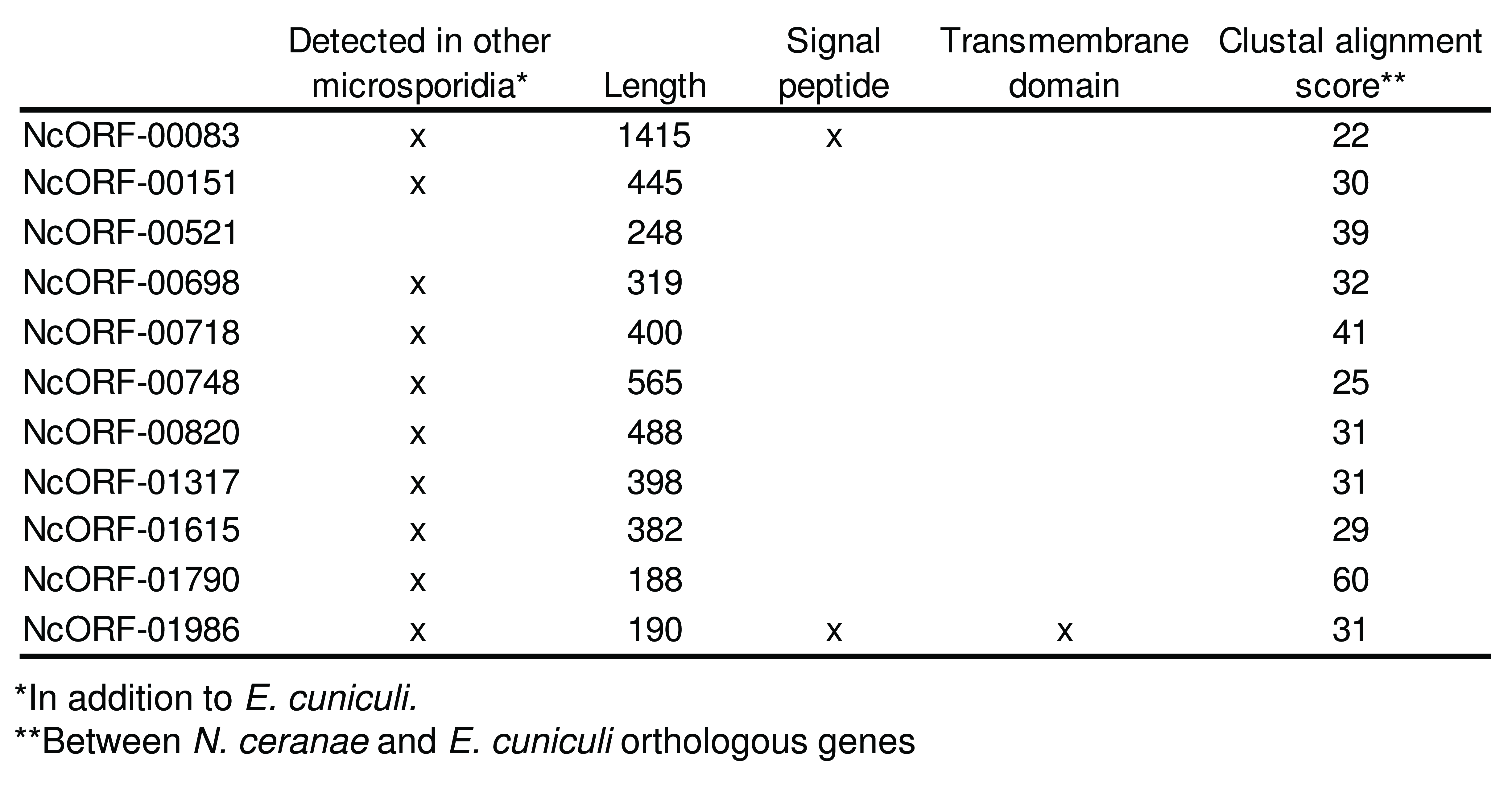

Supplement: Figure S10 — Characteristics of ‘microsporidian-specific’ genes, orthologous pairs of genes found in N. ceranae and E. cuniculi that lack apparent homology with proteins of taxa outside of order Microsporidia. (1.12 MB TIF) [file ppat.1000466.s015.tif]

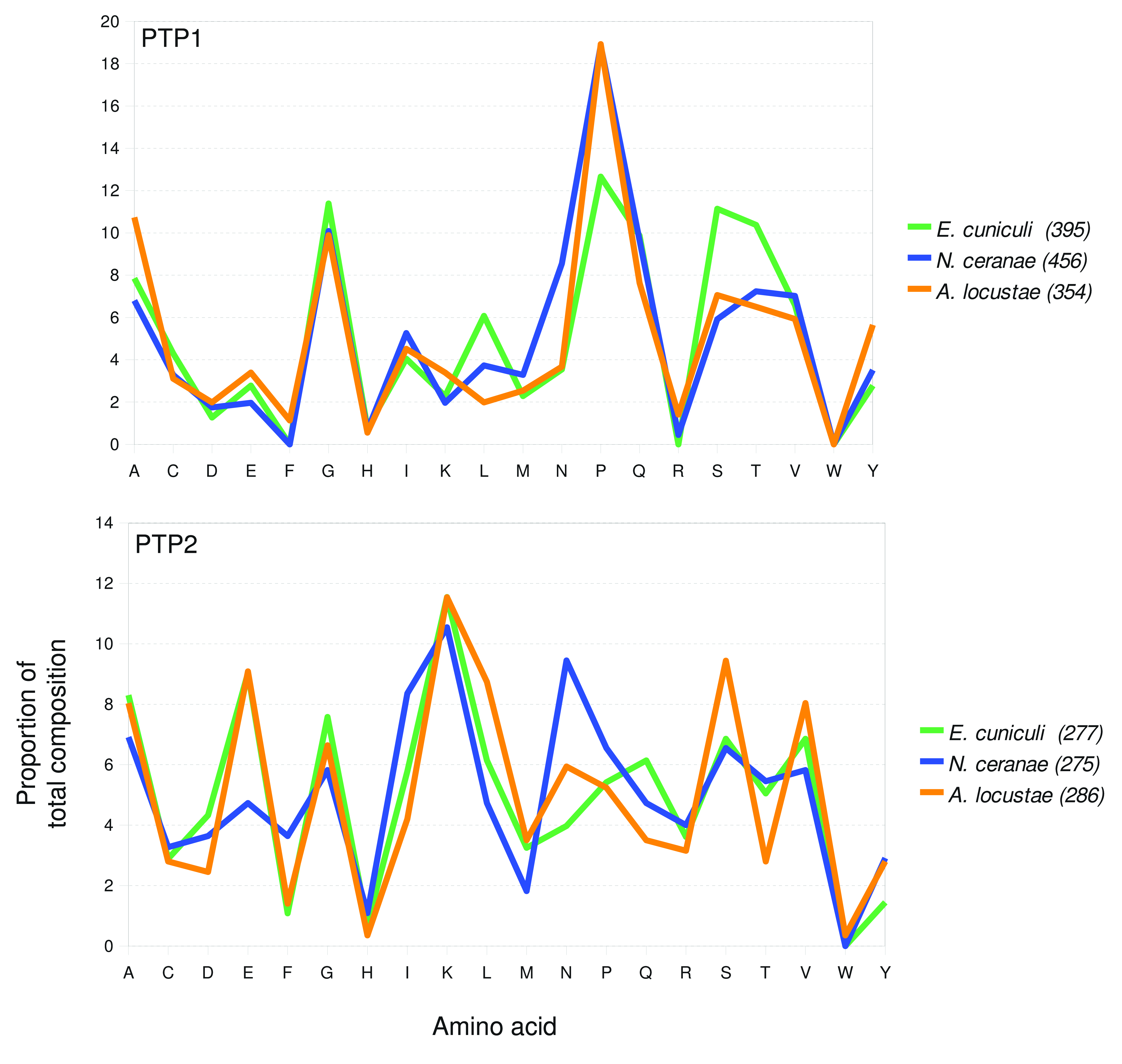

Supplement: Figure S11 — Amino-acid compositions of the putative polar tube proteins PTP1 and PTP2 in N. ceranae and two other microsporidians, E. cuniculi and A. locustae. Lengths of predicted proteins, in amino acid residues, is given in parentheses. (1.86 MB TIF) [file ppat.1000466.s016.tif]

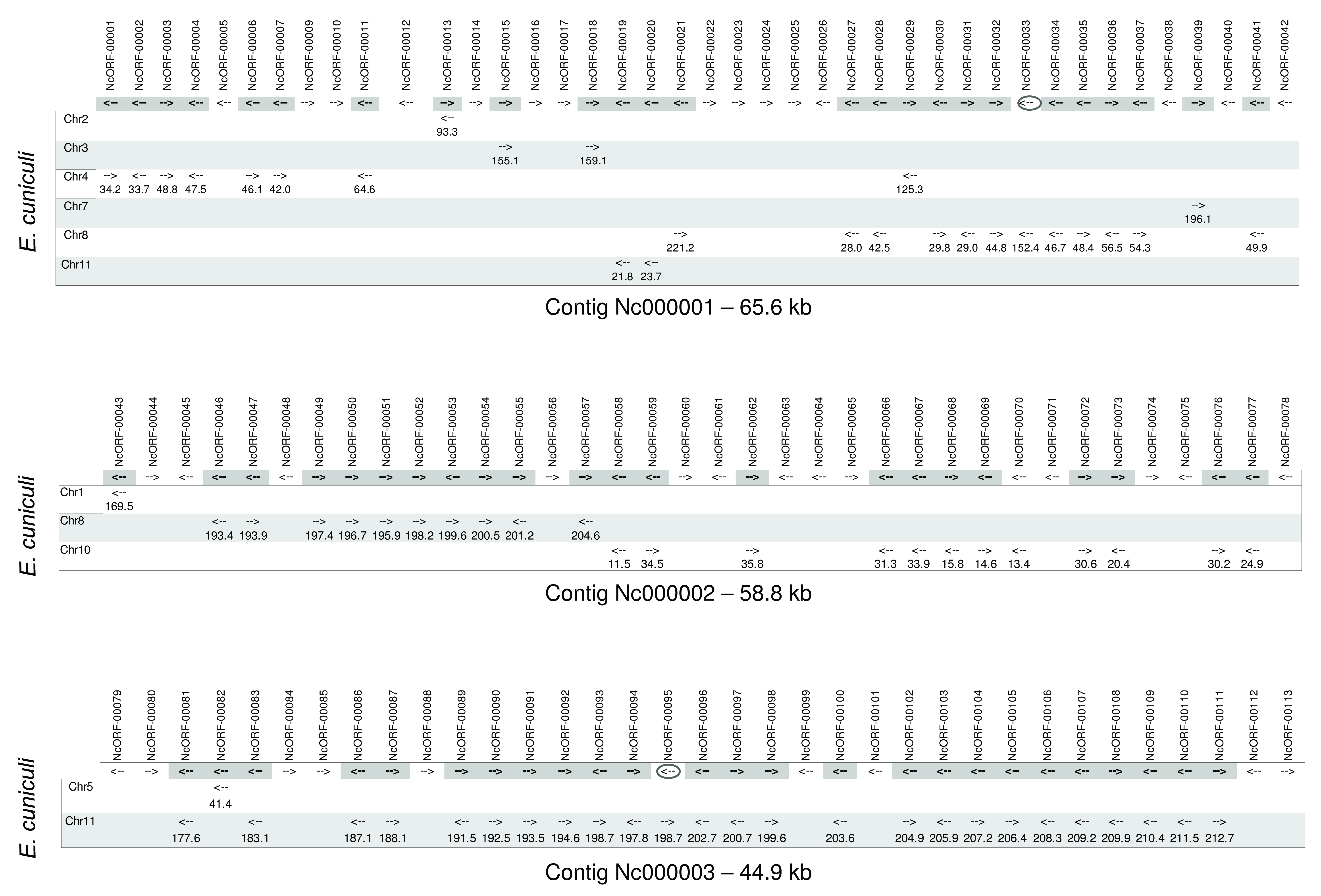

Supplement: Figure S12 — Degree of synteny between the N. ceranae contigs and E. cuniculi chromosomes. For each of the three largest contigs, predicted N. ceranae genes are shown in order along the contig (not to scale). The relative orientation of each gene is indicated by the arrow. N. ceranae genes shaded gray have one-to-one orthologs with E. cuniculi genes, whereas circled genes have homologs in E. cuniculi but not a one-to-one ortholog. Unmarked genes have no detected homolog in E. cuniculi (see text). The position in kilobases and relative orientation of the E. cuniculi ortholog is shown directly below the N. ceranae gene in the row corresponding to its chromosomal location. Coordinates are based on the GenBank record for each chromosome. These contigs contain regions of extensive, coarse-scale synteny with E. cuniculi, within which there can be considerable change in gene order or orientation. There are also numerous breaks in synteny associated with either a switch in E. cuniculi chromosome or an intervening, non-homologous gene. (2.06 MB TIF) [file ppat.1000466.s017.tif]

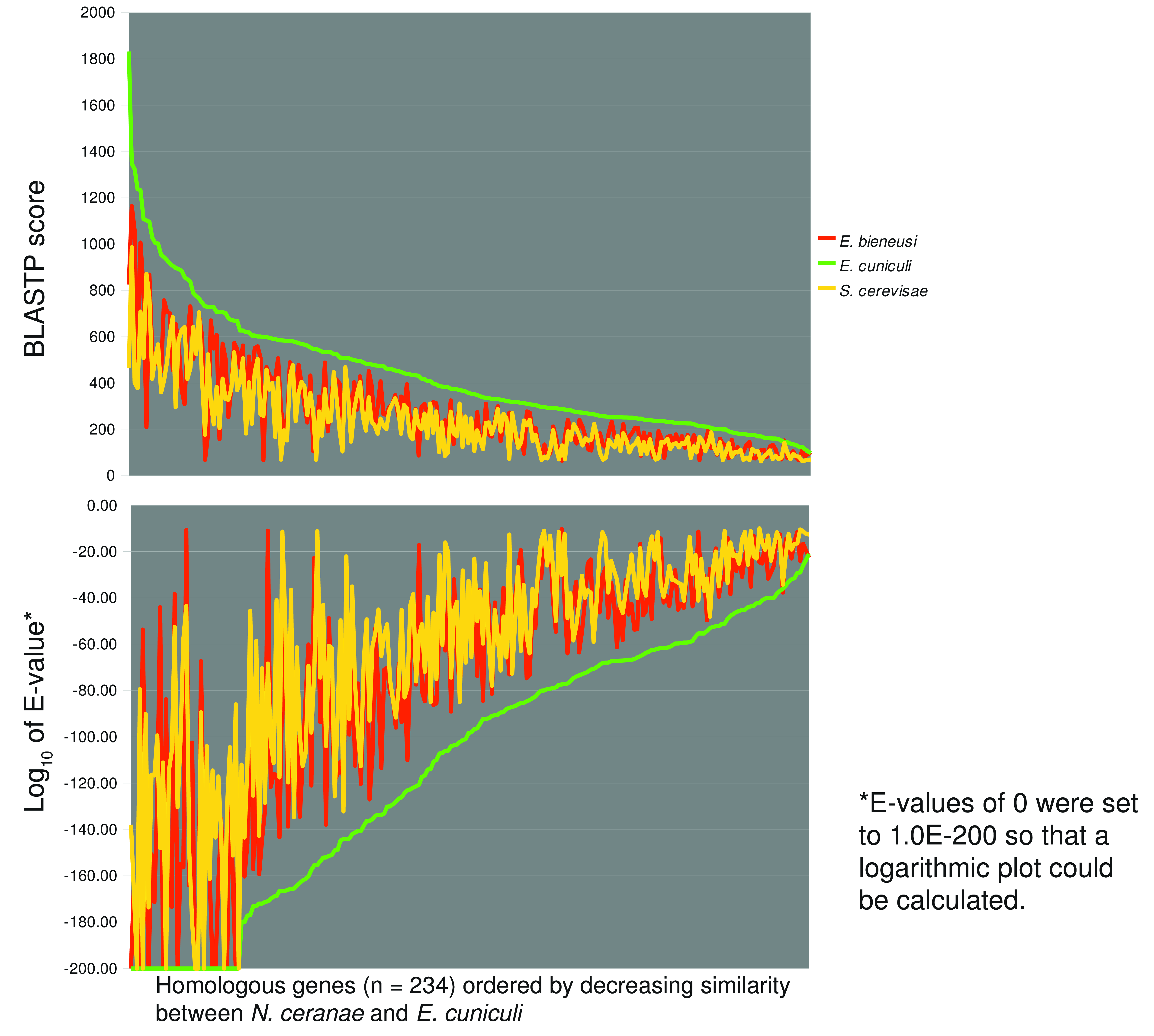

Supplement: Figure S13 — Relative sequence conservation between N. ceranae proteins and their homologs in other reference species. N. ceranae genes with one-to-one orthologs in E. cuniculi and yeast (see text) were BLASTP searched against the combined proteomes of E. cuniculi, E. bieneusi, and yeast. The number of N. ceranae genes with high-scoring matches in all three reference species in this data set was 234. The upper panel plots the BLASTP score of each N. ceranae gene versus the best match in each species, ordered along the X-axis by descending score versus E. cuniculi. Values are represented as lines rather than points for easier visualization. The lower panel plots the BLASTP expectation (E-value) in ascending order versus E. cuniculi. E-values equal to zero were set to 1.0E-200 to allow a logarithmic scale. (2.63 MB TIF) [file ppat.1000466.s018.tif]

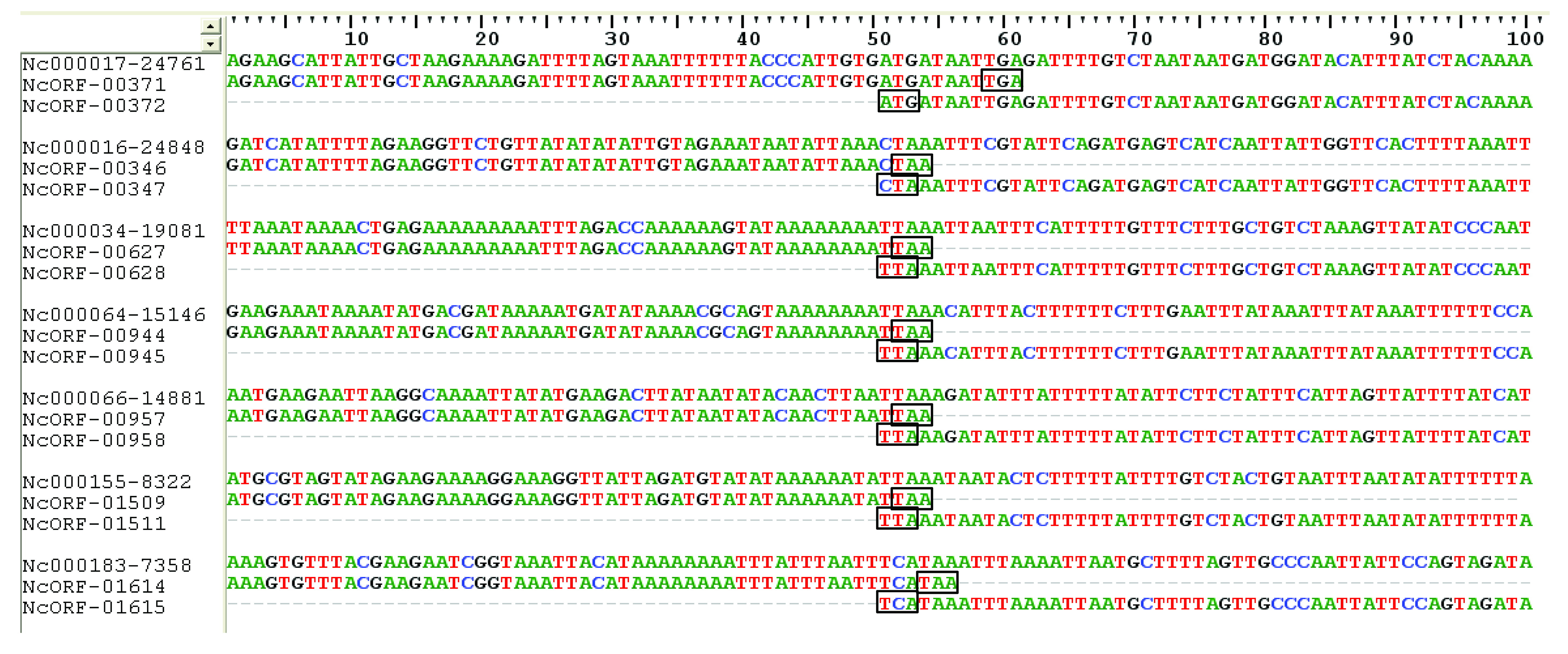

Supplement: Figure S14 — Alignment of adjacent N. ceranae genes that are supported by homology (see Results) and that overlap in sequence. Each set of three sequences represents a contig and two adjacent genes. Start and stop codons are indicated by boxes. (2.37 MB TIF) [file ppat.1000466.s019.tif]
